# p53 and fatty acids collaborate to trigger ferroptosis via the FBXO2-FABP5 axis in colorectal cancer

**DOI:** 10.1016/j.redox.2026.104043

**Published:** 2026-01-19

**Authors:** Jing Tong, Tao Han, Jun Deng, Yu Gan, Ruiwen Ruan, Wei Zhao, Chen Xiong, Quan Liao, Shiqi Chen, Huitong Bu, Jianping Xiong, Xiang Zhou, Qian Hao

**Affiliations:** a Cancer Institute, Fudan University Shanghai Cancer Center, Shanghai, 200032, China; b Department of Oncology, Shanghai Medical College, Fudan University, Shanghai, 200032, China; c Xinxiang Key laboratory for Molecular Oncology, Institutes of Health Central Plains, Henan Medical University, Xinxiang, 453003, China; d Department of Oncology, The First Affiliated Hospital, Jiangxi Medical College, Nanchang University, Nanchang, Jiangxi, 330006, China; e Jiangxi Key Laboratory for Individual Cancer Therapy, Nanchang, Jiangxi, 330006, China; f Key Laboratory of Breast Cancer in Shanghai, Department of Breast Surgery, Fudan University Shanghai Cancer Center, Shanghai, 200032, China

**Keywords:** p53, Ferroptosis, Polyunsaturated fatty acid (PUFA), Chaperone-mediated autophagy (CMA), Cancer therapy

## Abstract

In colorectal cancer (CRC), p53 can either suppress or potentiate tumor sensitivity to ferroptosis under oxidative stress conditions. However, it remains to be elucidated how p53 differentially regulates ferroptosis, and whether it can initiate ferroptosis. Our findings reveal that p53 induces ferroptosis in the presence of abundant polyunsaturated fatty acids (PUFAs). FBXO2, which is encoded by a p53-inducible target gene, interacts with FABP5 and promotes the lysosomal degradation of FABP5 through chaperone-mediated autophagy. This results in a decrease in the levels of PUFAs, thereby increasing resistance to ferroptosis in CRC. Notably, the supplementation of arachidonic acid not only reverses p53-mediated ferroptosis resistance, but also coordinates with p53 to initiate ferroptosis independently of additional oxidative stress, effectively suppressing the growth of CRC cells both in vitro and in vivo. Altogether, our study uncovers that the availability of PUFAs is crucial for p53 to exert a pro-ferroptotic function in CRC.

## 1 Introduction

Ferroptosis is orchestrated by three principal biochemical pathways: iron homeostasis, lipid peroxidation, and antioxidant system [[1], [2], [3], [4], [5]]. The tumor suppressor p53 plays a pivotal role in regulating cellular responses to ferroptosis inducers, such as Erastin and reactive oxygen species (ROS) [6,7]. The first evidence indicating that p53 modulates ferroptosis was reported in 2015 [8]. Solute carrier family 7 member 11 (SLC7A11), which is a component of the cystine/glutamate antiporter system x_c_^−^, is responsible for the cellular uptake of cystine and the consequent synthesis of glutathione (GSH) [9], a reductant that detoxifies ROS and prevents ferroptosis. p53 was found to repress the transcription of *SLC7A11*, thereby enhancing ferroptosis induced by Erastin or in response to elevated ROS levels [8,10,11]. Afterward, studies have demonstrated that p53 promotes ferroptosis via multiple mechanisms. For example, p53 increases the peroxidation of polyunsaturated fatty acids (PUFAs) by boosting the activity of arachidonate 12-lipoxygenase, 12S type (ALOX12) [12], and arachidonate 15-lipoxygenase (ALOX15) [13] upon ROS stress. Recently, p53 has been shown to inhibit the expression of vitamin K epoxide reductase complex subunit 1 like 1 (VKORC1L1), a potent ferroptosis suppressor that generates the reduced form of vitamin K [14]. In addition, p53 can sensitize cancer cells to ferroptosis by modulating mitochondrial activity [[15], [16], [17]], lipid metabolism [18], and iron homeostasis [19,20].

Under certain stress conditions, p53 can also contribute to defending ferroptosis. The p53 target gene cyclin dependent kinase inhibitor 1A (CDKN1A, also known as p21) was found to compete with KEAP1 for binding to NRF2, thereby enhancing NRF2 protein stability and protecting cells from oxidative stress [21]. Also, the activation of p21 leads to a delayed onset of ferroptosis under the condition of cystine deprivation [22]. In addition, p53 increases the expression of PLA2G6 to promote the detoxification of peroxidized lipids [23], or activates PLTP to facilitate the formation of lipid droplets, preventing the accumulation of peroxidized lipids in the plasma membrane [24]. It is hypothesized that p53 can help repair oxidative damage and avoid ferroptosis under mild stress. In contrast, when cells face extreme stress or irreversible lipid-peroxidation damage, p53 drives cell death by potentiating ferroptosis [6,7].

Recently, p53 has been found to inhibit ferroptosis in colorectal cancer (CRC) regardless of stress conditions. This is because p53 directly binds to dipeptidyl peptidase 4 (DPP4) and relocates it to the nucleus, thereby inhibiting DPP4-dependent lipid peroxidation [25]. However, several other studies have shown that the inhibition of p53 during chemotherapy results in the resistance to ferroptosis and a poor prognosis of patients with CRC, implying that p53 may increase ferroptosis sensitivity in CRC cells [26,27]. These seemingly contradictory findings suggest the existence of unknown mechanisms that govern the dual role of p53 in ferroptosis. In our study, we uncover that p53-mediated limitation of PUFA levels mitigates ferroptosis in CRC, despite its suppression of antioxidant pathways [8,14]. Our results identify the gene encoding F-box protein 2 (FBXO2) as a transcriptional target of p53. Surprisingly, FBXO2 acts as an oncogenic protein that fosters the growth and migration of cancer cells, even though its expression can be induced by p53. Mechanistic study reveals that FBXO2 binds to and promotes the lysosomal degradation of fatty acid binding protein 5 (FABP5) through the chaperon-mediated autophagy (CMA) pathway. The downregulation of FABP5 leads to a decrease in the levels of PUFAs, thus rendering resistance to ferroptosis in CRC cells. Remarkably, our results demonstrate that the supplementation of arachidonic acid (AA) not only reverses p53-mediated resistance to Erastin-induced ferroptosis but also synergizes with p53 to trigger ferroptosis and suppress CRC growth.

## 2 Results

### 2.1 FBXO2 is a transcriptional target of p53

**Fig. 1:**
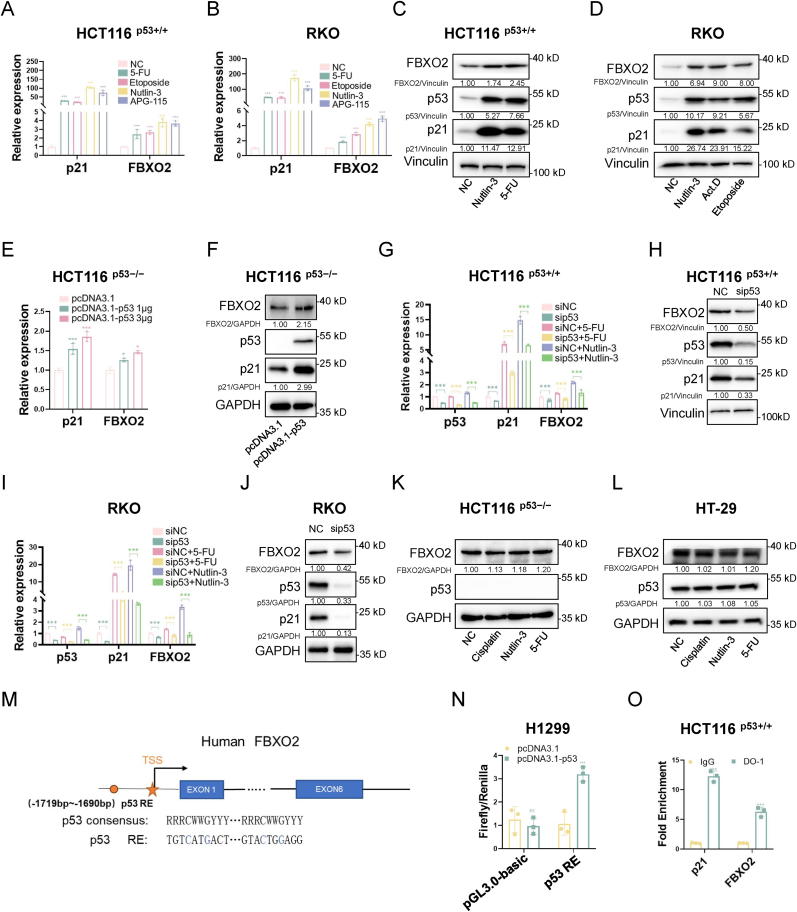
FBXO2 is a transcriptional target of p53. Data are represented as mean ± standard deviation (SD), *n* = 3. ∗*p* < 0.05, ∗∗∗*p* < 0.001, ns, not significant. See also Fig. S1. **(A and B)** qPCR analysis of the expression of FBXO2 mRNA in HCT116 ^p53+/+^ (A) and RKO (B) cells treated with or without 5-fluorouracil (5-FU, 40 μM), Etoposide (20 μM), Nutlin-3 (20 μM), or Alrizomadlin (APG-115, 2 μM) for 24 h **(C and D)** IB analysis of FBXO2 protein expression in HCT116 ^p53+/+^ (C) and RKO (D) cells treated with or without 5-FU, Etoposide, Nutlin-3, or Actinomycin D (Act.D) for 24 h **(E and F)** qPCR (E) and IB (F) analyses of FBXO2 expression in HCT116 ^p53−/−^ cells transfected with the indicated plasmids. **(G**–**J)** qPCR (G and I) and IB (H and J) analyses of FBXO2 expression in HCT116 ^p53+/+^ and RKO cells treated with the siRNAs and agents as indicated. **(K and L)** IB analysis of FBXO2 protein expression in HCT116 ^p53−/−^ (K) and HT-29 (with p53-R273H) (L) cells treated with or without the indicated agents. (M) The schematic of the potential p53-responsive element (p53-RE) on the FBXO2 promoter. (N) The luciferase reporter assay verifies the activation of the FBXO2 promoter by p53. (O) ChIP-qPCR analysis confirms the association of p53 with p53-RE on the promoter.

To comprehensively screen for novel p53 target genes, we analyzed our RNA-sequencing data from CRC HCT116 ^p53+/+^ cells treated with or without 5-Fluorouracil (5-FU) [28], the microarray data from breast cancer CAL51 cells treated with Cisplatin or Nutlin-3 [29,30], and the data of GSE201021 [31] as previously described. After a thorough evaluation, we found that *FBXO2* could be a potential transcriptional target gene of p53 (Fig. S1A–S1C). To confirm this observation, we treated wild-type p53-harboring cancer cells with different p53 agonists, including 5-FU, Etoposide, Nutlin-3, and Alrizomadlin (APG-115). Our results showed that the mRNA levels of FBXO2 were significantly elevated in multiple cancer cell lines with wild-type p53, including HCT116 ^p53+/+^, RKO, CAL51, MCF-7, and A549 (Fig. 1A,1B, S1D-S1F). Also, the protein levels of FBXO2 were increased upon p53 activation in both HCT116 ^p53+/+^ and RKO cells (Fig. 1C and D). In addition, both the mRNA and protein levels of FBXO2 were upregulated when wild-type p53 was overexpressed in HCT116 ^p53−/−^ cells (Fig. 1E and F). Conversely, the knockdown of p53 led to a reduction in the mRNA and protein levels of FBXO2 (Fig. 1G–J, S1G, S1H), which prevented the upregulation of FBXO2 levels induced by 5-FU, Nutlin-3, or Cisplatin. In contrast, these agents failed to induce the expression of FBXO2 in p53-null or -mutated cancer cells, including HCT116 ^p53−/−^, HT-29, H1299, TOV112D, and OVCA420 (Fig. 1K and L, S1I-S1L). These results suggest that FBXO2 expression might be transcriptionally upregulated by p53. To further validate this hypothesis, we employed the p53MH algorithm [32] to analyze the genomic sequence upstream of the transcription initiation site (TIS) of *FBXO2*. Through the analysis, we precisely identified a conserved p53-responsive element (p53-RE) located at −1719 to −1690 base pairs (Fig. 1M). Notably, the overexpression of p53 drastically induced the luciferase activity driven by the −1719 to −1690 region of *FBXO2* (Fig. 1N). Additionally, the chromatin immunoprecipitation (ChIP) assay further confirmed that p53 bound to this p53-RE in HCT116 ^p53+/+^ cells, with a p53-RE on the p21 promoter serving as a positive reference (Fig. 1O). Collectively, these results reveal that FBXO2 is a bona fide transcriptional target gene of p53 in various human cancer cells, including CRC cells.

### 2.2 FBXO2 promotes the growth and progression of colorectal cancer

**Fig. 2:**
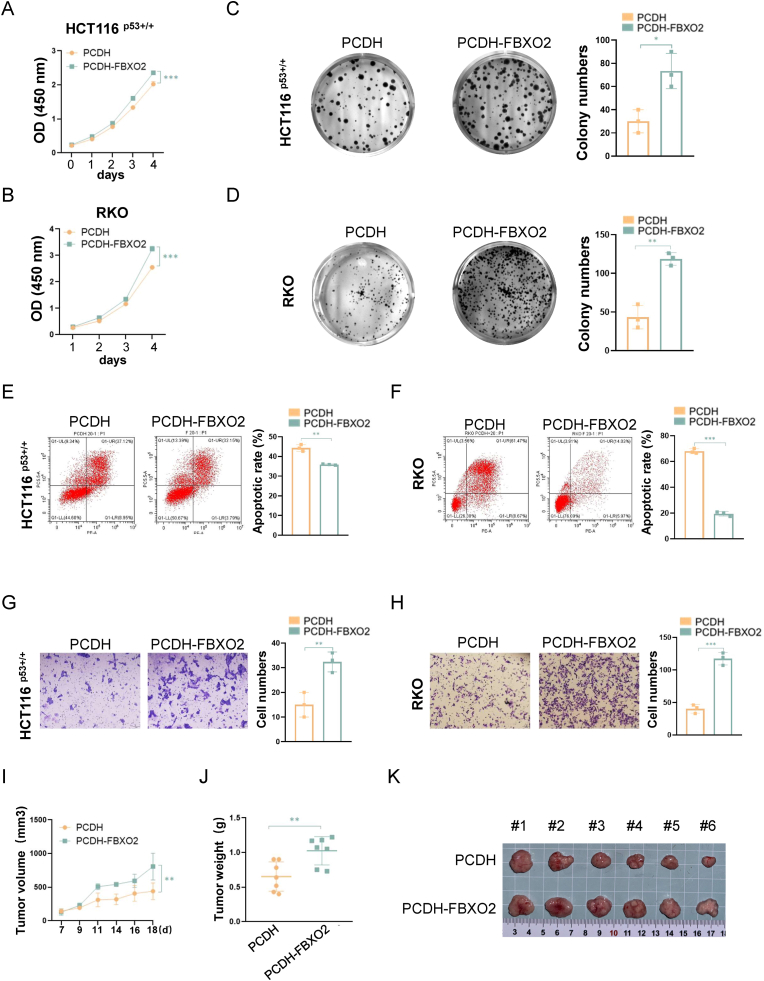
FBXO2 promotes the growth and progression of colorectal cancer Data in (I) and (J) are represented as mean ± SD, *n* = 6. ∗∗*p* < 0.01. See also Fig. S2. (A–H) HCT116 p53+/+ and RKO cells were transfected with an empty vector or PCDH-FBOX2, followed by the cell viability assay (A and B), colony formation assay (C and D), flow cytometric assay (E and F), and transwell migration assay (G and H). Data are represented as mean ± SD, *n* = 3. ∗*p* < 0.05, ∗∗*p* < 0.01, ∗∗∗*p* < 0.001. Scale bars, (G)1 mm, (H)100 μm **(I–K)** The growth rate (I), weight (J), and size (K) of xenograft tumors derived from HCT116 ^p53+/+^ cells stably overexpressing an empty vector or PCDH-FBXO2.

We then determined the biological function of FBXO2 in CRC cells. Firstly, we transfected the FBXO2-encoding plasmid into HCT116 ^p53+/+^ and RKO cells. To our surprise, the results showed that the overexpression of FBXO2 remarkably increased cell growth (Fig. 2A and B) and colony formation (Fig. 2C and D). In addition, the flow cytometric analysis showed that ectopic FBXO2 reduced apoptosis in CRC cells (Fig. 2E and F). Moreover, ectopic FBXO2 enhanced the migration of these CRC cells (Fig. 2G and H). Conversely, the knockdown of FBXO2 with two independent siRNAs significantly inhibited the growth (Fig. S2A and S2B), colony formation (Fig. S2C and S2D), and migration (Fig. S2G and S2H), while increasing apoptosis (Fig. S2E and S2F) in these CRC cells. The tumor-promoting role of FBXO2 was further validated in xenograft tumor models. In line with the cell-based results, stable overexpression of FBXO2 in HCT116 ^p53+/+^ cells significantly increased the growth rate (Fig. 2I), weight (Fig. 2J), and size (Fig. 2K) of xenograft tumors derived from these cells. In contrast, the depletion of FBXO2 markedly suppressed the growth of xenograft CRC tumors (Fig. S2I–S2K). Together, these results demonstrate that FBXO2 functions as an oncogenic protein in CRC, despite its activation by p53.

### 2.3 FBXO2 interacts with and destabilizes FABP5

**Fig. 3:**
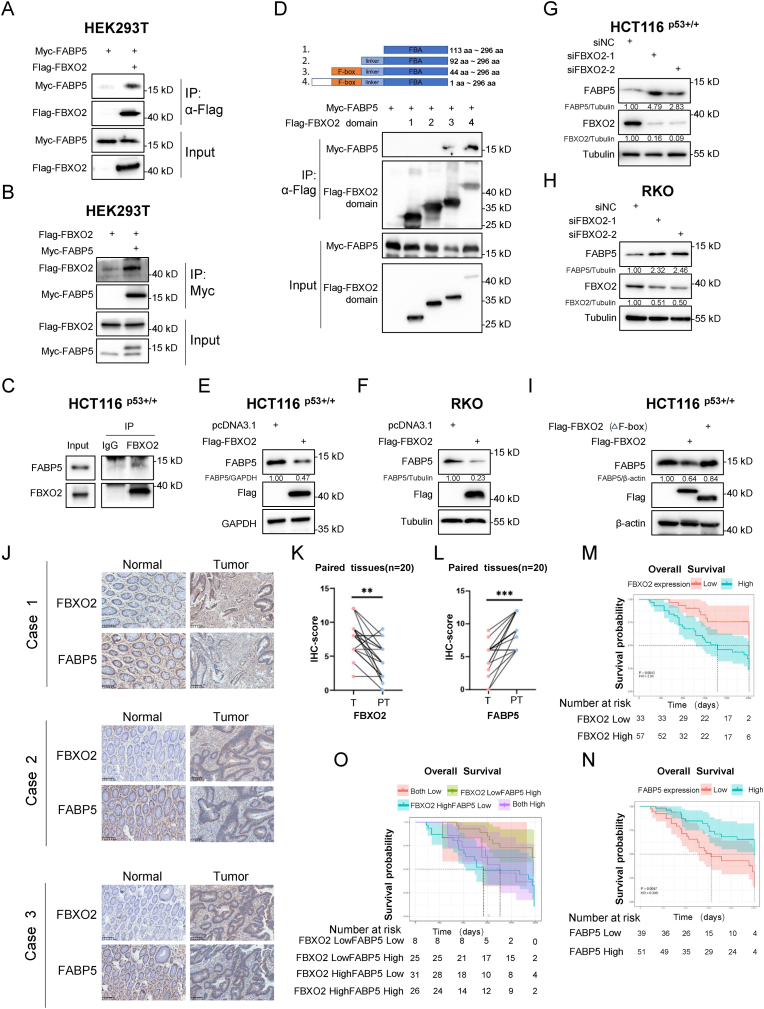
FBXO2 interacts with and destabilizes FABP5 in colorectal cancer **(A and B)** HEK293T cells were transfected with the indicated plasmids, followed by co-IP-IB analysis using antibodies as indicated. (C) HCT116 p53+/+ cells were treated with MG132 (20 μM) for 6 h, followed by co-IP-IB analysis. (D) HEK293T cells were transfected with the indicated plasmids, followed by co-IP-IB analysis. **(E and F)** IB analysis of FABP5 expression in HCT116 ^p53+/+^ (E) and RKO (F) cells transfected with the indicated plasmids. **(G and H)** IB analysis of FABP5 expression in HCT116 ^p53+/+^ (G) and RKO (H) cells transfected with the indicated siRNAs. **(I**) IB analysis of FABP5 expression in HCT116 ^p53+/+^ cells transfected with the indicated plasmids. (J) Representative images of IHC staining of FBXO2 and FABP5 in normal and colorectal cancer tissues. Scale bars, 100 μm **(K and L)** Statistical analysis of FBXO2 and FABP5 expression based on IHC scores in 20 pairs of matched colorectal tissues. ∗∗*p* < 0.01, ∗∗∗*p* < 0.001. (M) Higher levels of FBXO2 are associated with worse prognosis in 90 patients with CRC. (N) Higher levels of FABP5 are associated with better prognosis in 90 patients with CRC. (O) High levels of FBXO2 combined with low levels of FABP5 predict the worst prognosis in patients with CRC. See also Figs. S3 and S4.

To clarify the molecular mechanism underlying FBXO2's role in CRC, we performed co-immunoprecipitation (co-IP) in combination with mass spectrometry analysis, and identified that FABP5 could be a potential interacting protein of FBXO2. Since FABP5 was found to be involved in the regulation of ferroptosis by driving the redistribution of redox-sensitive lipids [33,34], we decided to verify whether FBXO2 binds to FABP5. Through reciprocal co-IP assays, we found that exogenously expressed FBXO2 and FABP5 indeed interacted with each other (Fig. 3A and B). In addition, the interaction between endogenous FBXO2 and FABP5 proteins were validated in HCT116 ^p53+/+^ cells (Fig. 3C). Immunofluorescence microscopy analysis indicated that the interaction between FBXO2 and FABP5 might occur in the cytoplasm of the cells (Fig. S3A). Then, we generated a series of deletion mutations of the FBXO2 protein and investigated which domain(s) of FBXO2 could bind to FABP5. Our results showed that FABP5 specifically bound to the N-terminus of FBXO2 (Fig. 3D), containing the F-box domain that is required for the binding to SKP1 and thus the formation of the SKP1–cullin1–F-box (SCF) ubiquitin ligase [35]. Subsequently, we explored whether FBXO2 regulated the protein level of FABP5. Immunoblotting (IB) analysis revealed that ectopic expression of FBXO2 led to a decrease in FABP5 protein levels (Fig. 3E and F), while the knockdown of FBXO2 resulted in an increase in FABP5 protein levels (Fig. 3G and H). We also found that the F-box was critical for FBXO2-mediated downregulation of FABP5, as FBXO2 with the deletion of the F-box domain had no impact on the protein level of FABP5 (Fig. 3I). Since FBXO2 expression is transcriptionally activated by p53, we tested whether p53 could reduce FABP5 levels through FBXO2. As anticipated, activation of p53 using Nutlin-3, APG-115, or 5-FU decreased the protein levels of FABP5 in a dose-dependent manner in CRC cells (Fig. S3B–S3G). Consistently, in p53-null HCT116 ^p53−/−^ and H1299 cancer cells, ectopic expression of p53 markedly reduced FABP5 protein levels (Fig. S3H and S3I). In contrast, Nutlin-3 treatment had no impact on the FABP5 protein level in HCT116 ^p53−/−^ cells (Fig. S3J). Together, these results demonstrate that p53-inducible FBXO2 binds to FABP5, thereby reducing FABP5 protein levels.

### 2.4 FBXO2 and FABP5 are inversely expressed in colorectal cancer

As described above, the overexpression of FBXO2 reduces FABP5 protein levels in CRC cells. Hence, we tested whether there was a correlation between the expression of the two proteins in CRC samples. First, we detected their expression in CRC tissues and paired normal tissues by immunohistochemistry (IHC) as represented in Fig. 3J. The results showed FBXO2 was expressed at higher levels in CRC than in normal tissues (Fig. 3K), whereas FABP5 was expressed at lower levels in CRC than in normal tissues (Fig. 3L). In addition, by evaluating the expression of the two proteins in CRC tissues from a cohort of 90 patients, we found that the higher expression of FBXO2 was significantly associated with worse overall survival of patients with CRC (Fig. 3M). On the contrary, higher expression of FABP5 was significantly associated with better prognosis (Fig. 3N). Our analysis further revealed an inverse correlation between FBXO2 and FABP5 expression in CRC tissues (Fig. S3K). Remarkably, a high level of FBXO2 combined with a low level of FABP5 predicted the worst prognosis in patients with CRC (Fig. 3O). Moreover, both univariate and multivariate analyses indicated that FBXO2 and FABP5 could serve as prognostic factors in CRC (Fig. S3L and Table S1). Consistently, analyses of TCGA and CPTAC databases revealed that FBXO2 expression levels were higher, while FABP5 expression levels were lower, in CRC tissues compared with normal tissues (Fig. S4A and S4B). Additionally, elevated FBXO2 or reduced FABP5 levels were associated with worse recurrence-free survival (Fig. S4C and S4D). Together, these results highlight the clinical significance of FBXO2 and FABP5 in CRC and suggest that FBXO2-mediated downregulation of FABP5 may contribute to poorer patient survival in the disease.

### 2.5 FBXO2 promotes FABP5 degradation through chaperon-mediated autophagy

**Fig. 4:**
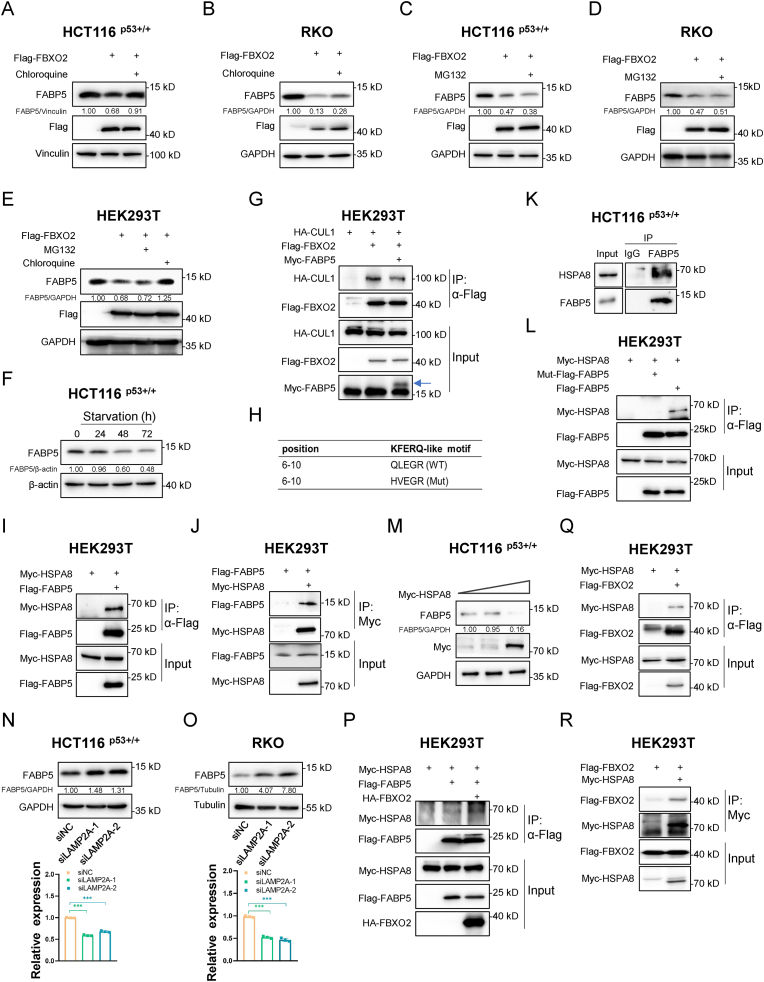
FBXO2 promotes FABP5 degradation through chaperon-mediated autophagy. **(A**–**D)** HCT116 ^p53+/+^ and RKO cells were transfected with an empty vector or Flag-FBXO2, and then treated with either Chloroquine (10 μM) for 8 h (A and B) or MG132 (20 μM) for 6 h (C and D), followed by IB analysis using antibodies as indicated. (E) HEK293T cells were transfected with an empty vector or Flag-FBXO2, and then treated with either Chloroquine or MG132, followed by IB analysis. (F) HCT116 p53+/+ cells were cultured in serum-free medium for different time intervals, followed by IB analysis. (G) HEK293T cells were transfected with the indicated plasmids, followed by co-IP-IB analysis. (H) A KFERQ-like motif, QLEGR, in FABP5 and the mutation of the motif to HVEGR. **(I and J)** HEK293T cells were transfected with the indicated plasmids, followed by co-IP-IB analysis. (K) HCT116 p53+/+ cells were collected and lysed for co-IP-IB analysis. (L) HEK293T cells were transfected with the indicated plasmids, followed by co-IP-IB analysis. (M) HCT116 p53+/+ cells were transfected with the indicated plasmids, followed by IB analysis. **(N and O)** HCT116 ^p53+/+^ and RKO cells were transfected with the indicated siRNAs, followed by IB analysis. The knockdown efficiency was verified by qPCR. (P) HEK293T cells were transfected with the indicated plasmids, followed by co-IP-IB analysis. **(Q and R)** HEK293T cells were transfected with the indicated plasmids, followed by co-IP-IB analysis.

Two major protein degradation systems in eukaryotic cells involve the ubiquitin-proteasome system and autophagy-lysosome system. To figure out the mechanism underlying FBXO2-mediated FABP5 degradation, we treated cells with the proteasome inhibitor MG132 or the autophagy inhibitor chloroquine (CQ). Our results revealed that FBXO2-induced degradation of FABP5 could be partially reversed by CQ (Fig. 4A and B), but not MG132 (Fig. 4C and D) in CRC cells. Consistently, these results were also validated in HEK293T cells (Fig. 4E). In addition, serum starvation-induced autophagy repressed the levels of FABP5 in a time-dependent fashion (Fig. 4F). These findings suggested that FBXO2 triggered FABP5 degradation through the autophagy-lysosomal pathway, but not the proteasomal pathway. Since FABP5 binds to the F-box domain of FBXO2 (Fig. 3D), a domain that is essential for the formation of the SCF E3 ligase complex [35], we wondered whether the binding of FABP5 perturbed the formation of the SCF E3 ligase complex. As shown in Fig. 4G, ectopic expression of FABP5 indeed impaired the interaction between FBXO2 and CUL1, a scaffold protein in the SCF complex. This result elucidates why FBXO2 fails to promote the proteasomal degradation of FABP5, as the interaction between FBXO2 and FABP5 prevents the formation of SCF E3 ligase complex. Importantly, we identified a KFERQ-like motif, QLEGR, in FABP5 (Fig. 4H), which is essential for the binding to HSPA8 and thus chaperone-mediated autophagy (CMA), a selective pathway for lysosomal proteolysis [[36], [37], [38]]. A substrate with a KFERQ-like motif forms a complex with the chaperone protein HSPA8. This complex is then translocated into lysosomes, facilitated by the lysosome membrane protein LAMP2A, for degradation [37]. Therefore, we tested whether FABP5 interacted with HSPA8. Reciprocal co-IP assays showed that ectopically expressed FABP5 and HSPA8 could bind to each other (Fig. 4I and J). In addition, the interaction between endogenous proteins was verified in CRC cells (Fig. 4K). Our result further revealed that the QLEGR motif was critical for this interaction, as mutation of QLEGR to HVEGR completely abolished the binding of FABP5 with HSPA8 (Fig. 4H and L). Furthermore, the overexpression of HSPA8 led to a dramatic decrease in the level of FABP5 (Fig. 4M), while the knockdown of LAMP2A resulted in an increase in FABP5 levels (Fig. 4N and O). Finally, our results showed that ectopic FBXO2 enhanced the interaction between FABP5 and HSPA8 (Fig. 4P). Interestingly, we also detected an interaction between FBXO2 and HSPA8 (Fig. 4Q and R). While this interaction might be attributed to an indirect linkage mediated by FABP5, our result suggested a crucial role of FBXO2 in facilitating the interaction between FABP5 and HSPA8. Together, these results demonstrate that FBXO2 promotes the degradation of FABP5 through the CMA pathway.

### 2.6 FABP5 enhances ferroptosis sensitivity by maintaining the levels of polyunsaturated fatty acids

**Fig. 5:**
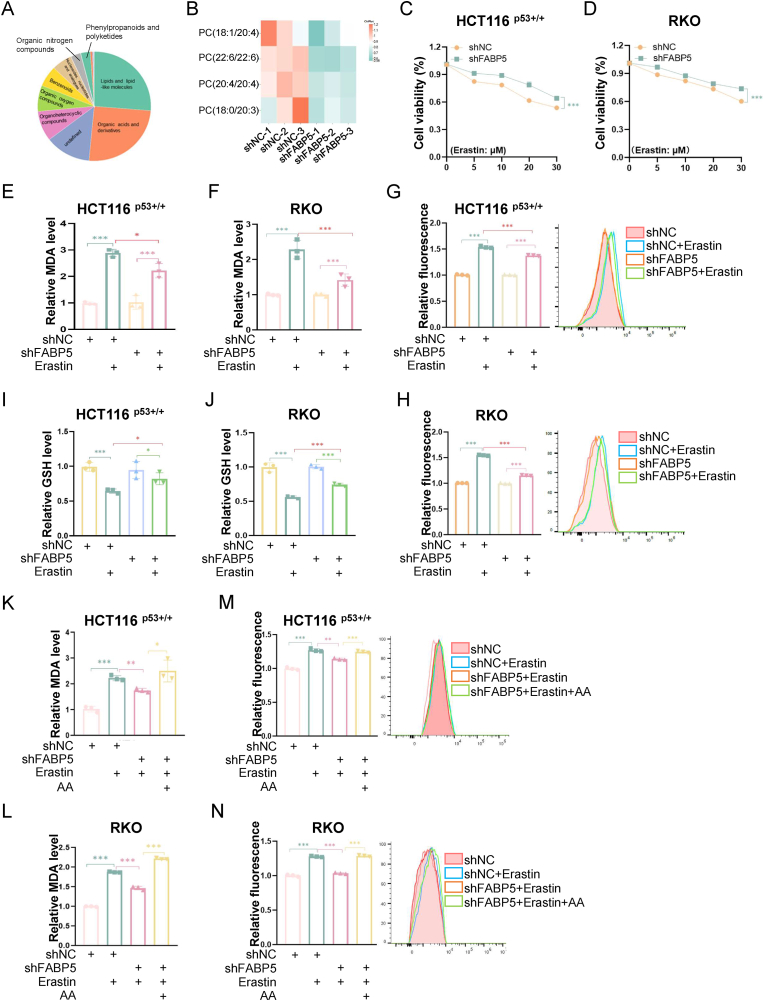
FABP5 enhances ferroptosis sensitivity by regulating levels of polyunsaturated fatty acids. Data are represented as mean ± SD, *n* = 3. ∗*p* < 0.05, ∗∗*p* < 0.01, ∗∗∗*p* < 0.001. See also Fig. S5. **(A)** Lipidomic analysis of HCT116 ^p53+/+^ cells stably expressing control (shNC) or FABP5 shRNA (shFABP5). **(B)** The levels of PUFA-containing phosphatidylcholines (PCs) in HCT116 ^p53+/+^ cells stably expressing shNC or shFABP5 revealed by lipidomic analysis. **(C and D)** HCT116 ^p53+/+^ (C) and RKO (D) cells stably expressing shNC or shFABP5 were exposed to different doses of Erastin for 24 h, followed by the cell viability assay. **(E and F)** HCT116 ^p53+/+^ (E) and RKO (F) cells stably expressing shNC or shFABP5 were treated with Erastin as indicated, followed by the MDA assay. **(G and H)** HCT116 ^p53+/+^ (G) and RKO (H) cells stably expressing shNC or shFABP5 were treated with Erastin as indicated, followed by the BODIPY™ 581/591C11 assay. **(I and J)** HCT116 ^p53+/+^ (I) and RKO (J) cells stably expressing shNC or shFABP5 were treated with Erastin as indicated, followed by the GSH assay. **(K and L)** HCT116 ^p53+/+^ (K) and RKO (L) cells stably expressing shNC or shFABP5 were treated with Erastin or AA as indicated, followed by the MDA assay. **(M and N)** HCT116 ^p53+/+^ (M) and RKO (N) cells stably expressing shNC or shFABP5 were treated with Erastin or AA as indicated, followed by the BODIPY™ 581/591C11 assay.

It has been known that FABP5 promotes the intracellular transport and metabolism of fatty acids across multiple cell-based and animal models [33,34,39]. Hence, we comprehensively examined whether FABP5 played a role in lipid metabolism in HCT116 ^p53+/+^ cells by conducting an untargeted lipidomic analysis. Our results revealed that the knockdown of FABP5 markedly reduced the levels of PUFAs, including arachidonic acid (AA, 20:4), docosahexaenoic acid (DHA, 22:6), and eicosatrienoic acid (ETA, 20:3) (Fig. 5A and B). Since PUFAs can serve as both the substrate and the driver of ferroptosis [[1], [2], [3], [4]], we tested whether FABP5 was associated with the susceptibility of CRC cells to ferroptosis. Through the cell viability assay, we found that ectopic expression of FABP5 significantly enhanced the sensitivity of CRC cells to Erastin (Fig. S5A and S5B). In contrast, the depletion of FABP5 partially alleviated Erastin- or RSL3-induced inhibition of cell growth (Fig. 5C and D, S5C, S5D). We also determined the levels of malondialdehyde (MDA), an end-product of lipid peroxidation. The levels of MDA, as expected, were dramatically elevated upon Erastin treatment. This elevation could be further augmented by the overexpression of FABP5 (Fig. S5E and S5F), yet hampered by the knockdown of FABP5 (Fig. 5E and F). In addition, the flow cytometric analysis was performed to evaluating BODIPY™ 581/591C11, a fluorescent probe for the detection of lipid ROS. Our results showed that the depletion of FABP5 significantly impeded Erastin-induced generation of lipid ROS in both HCT116 ^p53+/+^ and RKO cells (Fig. 5G and H). The antioxidant GSH cooperates with GPX4 to promote the removal of ROS, thereby inhibiting ferroptosis. Consequently, the level of GSH negatively correlates with the degree of ferroptosis. By evaluating the levels of GSH, we found that the overexpression of FABP5 promoted Erastin-induced reduction of GSH levels (Fig. S5G and S5H), whereas the knockdown of FABP5 partially counteracted the reduction of GSH levels (Fig. 5I and J). Given that the depletion of FABP5 resulted in the decline in PUFA levels (Fig. 5A and B), we questioned whether the supplementation of AA could reverse the ferroptosis resistance in CRC cells with depleted FABP5. Our results revealed that the knockdown of FABP5 reduced MDA levels upon Erastin treatment, while these effects were reversed by the addition of AA (Fig. 5K and L). Additionally, these results were further confirmed by the flow cytometric analysis of lipid ROS using the C11 probe (Fig. 5M and N). Together, these findings demonstrate that FABP5 facilitates ferroptosis by maintaining the levels of PUFAs.

Given that FBXO2 promoted the degradation of FABP5 (Fig. 3, Fig. 4), we were curious to know whether FBXO2 played a role in ferroptosis resistance in CRC cells. Our results revealed that the overexpression of FBXO2 mitigated the Erastin- or RSL3-induced inhibition of cell growth (Fig. S6A–S6D) and the elevation of MDA levels (Fig. S6E and S6F). On the contrary, the knockdown of FBXO2 enhanced the sensitivity of CRC cells to Erastin, as demonstrated by an additional reduction in cell growth (Fig. S6G and S6H) and a further elevation in MDA levels (Fig. S6I and S6J) in FBXO2-depleted cells. Therefore, these findings demonstrate that FBXO2 endows CRC cells with ferroptosis resistance, potentially by promoting the degradation of FABP5.

### 2.7 p53 promotes ferroptosis resistance via the FBXO2-FABP5 axis in colorectal cancer

**Fig. 6:**
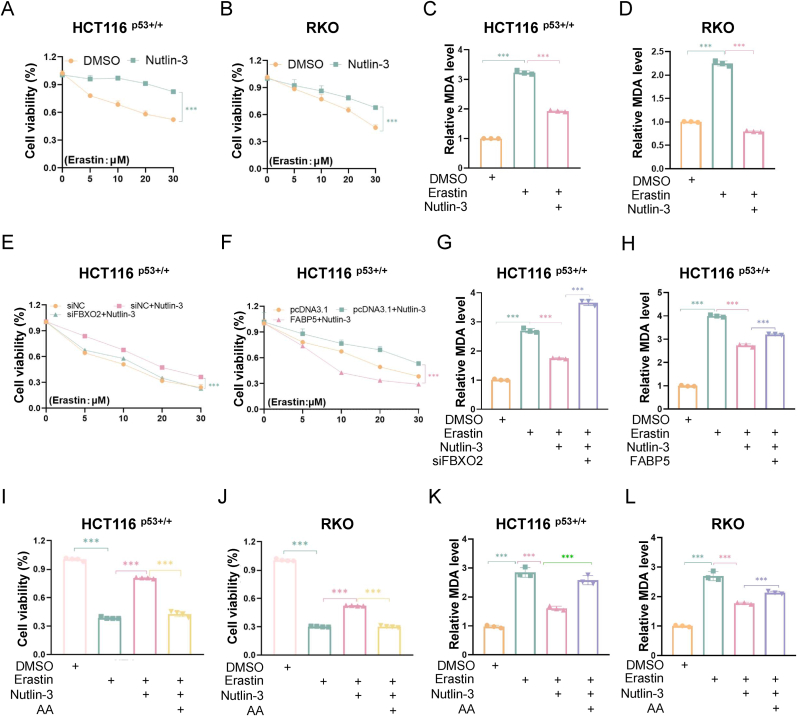
p53 promotes ferroptosis resistance via the FBXO2-FABP5 axis. Data are represented as mean ± SD, *n* = 3. ∗∗∗*p* < 0.001. See also Fig. S6. **(A and B)** HCT116 ^p53+/+^ (A) and RKO (B) cells were treated with DMSO, Nutlin-3, and Erastin as indicated for 48 h, followed by the cell viability assay. **(C and D)** HCT116 ^p53+/+^ (C) and RKO (D) cells were treated with DMSO, Nutlin-3, and Erastin as indicated for 48 h, followed by the MDA assay. **(E and F)** HCT116 ^p53+/+^ cells were transfected with the siRNAs (E) or plasmids (F), and treated with DMSO, Nutlin-3, and Erastin as indicated for 48 h, followed by the cell viability assay. **(G and H)** HCT116 ^p53+/+^ cells were transfected with the siRNAs (G) or plasmids (H), and treated with DMSO, Nutlin-3, and Erastin as indicated for 48 h, followed by the MDA assay. **(I and J)** HCT116 ^p53+/+^ (I) and RKO (J) cells were treated with the combinations of the indicated agents for 48 h, followed by the cell viability assay. **(K and L)** HCT116 ^p53+/+^ (K) and RKO (L) cells were treated with the combinations of the indicated agents for 48 h, followed by the MDA assay.

Next, we investigated whether p53 induced ferroptosis resistance in CRC cells and, if so, whether the FBXO2-FABP5 axis was responsible for mediating this process. The activity of p53 is determined and modulated through post-translational modifications in the context of various cellular stress signals [40,41]. To preclude the impact of cellular stress on p53'role in regulating ferroptosis, we utilized Nutlin-3 to purely activate p53 without affecting its phosphorylation or acetylation. The cell viability assay was performed to show that the activation of p53 indeed conferred resistance to ferroptosis in CRC cells (Fig. 6A, 6B, S6K, S6L). In addition, Nutlin-3 treatment repressed Erastin-induced production of MDA (Fig. 6C and D). These results demonstrate that p53 predominantly inhibits ferroptosis in CRC, which is in line with a previous study [25]. Then, we examined whether p53 induced ferroptosis resistance via the FBXO2-FABP5 axis. Our findings indicated that Nutlin-3 treatment partially alleviated the Erastin-induced inhibition of cell growth, whereas both the knockdown of FBXO2 and the overexpression of FABP5 could restore the effect of Erastin on cell growth (Fig. 6E and F). Consistently, the knockdown of FBXO2 or the overexpression of FABP5 was able to restore the Erastin-induced MDA generation during Nutlin-3 treatment (Fig. 6G and H). Furthermore, we hypothesized that p53-mediated resistance to ferroptosis might be associated with the reduction of PUFAs. Consequently, we tested whether the supplementation of AA would counteract this resistance. Our results revealed that the addition of AA indeed reversed Nutlin-3-induced ferroptosis resistance, as evidenced by the reduced cell growth (Fig. 6I and J) and elevated MDA levels (Fig. 6K and L) in the presence of exogenous AA. Therefore, these results demonstrate that p53 confers resistance to ferroptosis via the FBXO2-FABP5 axis in CRC.

### 2.8 Arachidonic acid cooperates with p53 to trigger ferroptosis

p53 facilitates lipid peroxidation by repressing antioxidant genes, such as *SLC7A11* [8] and *VKORC1L1* [14], or by activating ALOX12 [12] and ALOX15 [13] that catalyze lipid peroxidation. We also validated that Nutlin-3 treatment upregulated the expression of ACSL4, LPCAT3, ALOX15, and ALOX12, but downregulated SLC7A11 in CRC cells (Fig. S7A–S7D), with p21 expression monitored to confirm p53 activation. However, the p53-FBXO2-FABP5 axis reduces the levels of PUFAs, which are the essential substrates for lipid peroxidation. Consequently, this limitation of substrate availability is critical for p53-mediated ferroptosis resistance. Under this scenario, we postulated that AA supplementation, which counteracted the role of the FBXO2-FABP5 axis, could cooperate with p53 to induce ferroptosis. To verify this hypothesis, we conducted a combined treatment of CRC cells with Nutlin-3 and AA. Our results showed that the combination of Nutlin-3 with AA could more dramatically inhibit CRC cell growth (Fig. S7E and S7F) and elevate the level of lipid ROS (Fig. S7G and S7H), although the application of AA alone exerted a negligible effect on both cell growth and the ROS level. Given that p53 is a potent inducer of apoptosis, we asked whether the combination of Nutlin-3 and AA triggers ferroptosis, apoptosis, or both. The combined treatment increased PUMA expression but decreased SLC7A11 levels (Fig. S7I), suggesting both processes were activated. Interestingly, the treatment slightly elevated the levels of GPX4 (Fig. S7J), which might be an adaptive response, although GPX4 modulation is dispensable for p53-mediated ferroptosis [12]. Consistent with this, both ferroptosis and apoptosis inhibitors partially rescued cell viability (Fig. S7K and S7L). Furthermore, the ferroptosis inhibitors significantly suppressed lipid ROS and MDA production induced by the combination treatment (Fig. S7M–S7P), demonstrating the induction of ferroptosis.

**Fig. 7:**
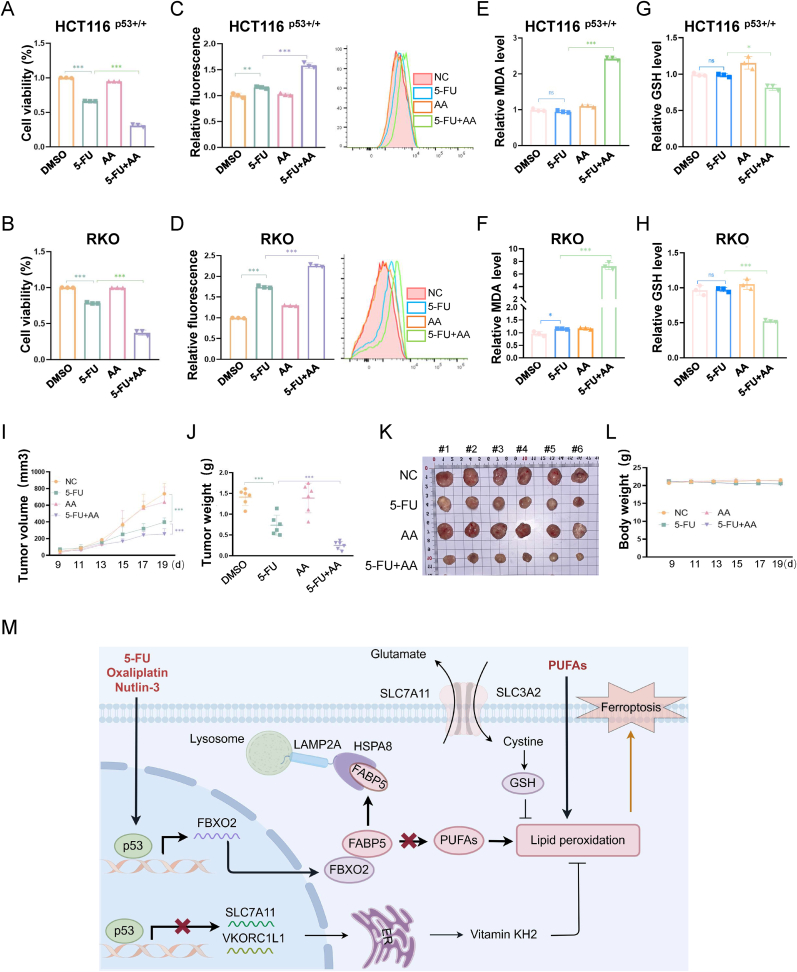
Arachidonic acid cooperates with p53 to trigger ferroptosis **(A and B)** HCT116 ^p53+/+^ (A) and RKO (B) cells were treated with the indicated agents for 48 h, followed by the cell viability assay. **(C and D)** HCT116 ^p53+/+^ (C) and RKO (D) cells were treated with the indicated agents for 48 h, followed by the BODIPY™ 581/591C11 assay. **(E and F)** HCT116 ^p53+/+^ (E) and RKO (F) cells were treated with the indicated agents for 48 h, followed by the MDA assay. **(G and H)** HCT116 ^p53+/+^ (G) and RKO (H) cells were treated with the indicated agents for 48 h, followed by the GSH assay. Data are represented as mean ± SD, *n* = 3. ∗*p* < 0.05, ∗∗*p* < 0.01, ∗∗∗*p* < 0.001, ns, not significant. See also Fig. S7. **(I**–**L)** The growth rate (I), weight (J), and size (K) of xenograft tumors derived from HCT116 ^p53+/+^ cells and treated with the indicated agents. Data in (I) and (J) are represented as mean ± SD, *n* = 6. ∗∗∗*p* < 0.001. (M) A working model depicts the cooperation of p53 and PUFAs in ferroptosis regulation. See also Fig. S7.

5-FU and Oxaliplatin, which are first-line chemotherapeutic drugs for CRC, can trigger p53 activation through DNA damage stress. We tested whether the combination of 5-FU or Oxaliplatin with AA could induce ferroptosis. First, our results indicated that the combined treatments more prominently suppressed the growth of both HCT116 ^p53+/+^ and RKO cells (Fig. 7A, 7B, S8A, S8B). In addition, the combined treatments elevated the levels of lipid ROS more markedly than single treatments did (Fig. 7C and D, S8C, S8D). Moreover, while single treatments had a marginal effect on the levels of MDA that is one of the final products of PUFA peroxidation, the combination of 5-FU with AA strikingly augmented MDA generation, indicating an increase in ferroptosis (Fig. 7E and F). Interestingly, we observed that 5-FU alone could moderately increase lipid ROS levels, while not influencing the levels of MDA (Fig. 7C–F). This could be attributed to the fact that the approach of detecting lipid ROS using the C11 probe is highly sensitive, and the detectable ROS generated by single 5-FU treatment is insufficient to trigger extensive lipid peroxidation, and consequently MDA generation and ferroptosis. This observation is also in line with our previous study [11]. Furthermore, the combination of 5-FU with AA markedly reduced the levels of GSH when compared with single treatments (Fig. 7G and H). Therefore, the above results demonstrate that 5-FU and AA can coordinately suppress the growth of CRC, in part, by triggering ferroptosis. Finally, to translate these findings into potential clinical relevance, we tested the anti-tumor activity of the combined treatment in a xenograft mouse model. Nude mice were subcutaneously inoculated with HCT116 ^p53+/+^ cells and then randomly assigned into four groups. These groups were treated with saline, 5-FU, AA, and a combination of 5-FU with AA, respectively. Our results revealed that single administration of a low dose of 5-FU moderately reduced the growth rate (Fig. 7I), weight (Fig. 7J), and size (Fig. 7K) of xenograft tumors. Remarkably, the combination of 5-FU with AA suppressed tumor growth to a significantly greater extent compared with individual treatments (Fig. 7I–K). The potential adverse effects were tolerable since the body weights of mice in each group were comparable (Fig. 7L). Collectively, these results demonstrate that the activation of p53 induced by, for example, Nutlin-3, Oxaliplatin, and 5-FU can coordinate with AA to suppress the growth of CRC by triggering ferroptosis.

## 3 Discussion

The dual role of p53 in orchestrating ferroptosis across human cancers, particularly in CRC, has been an intriguing area of investigation, as multiple studies with seemingly contradictory evidence report that p53 differentially modulates ferroptosis sensitivity in response to inducers (e.g., Erastin) or ROS stress [6,7,[25], [26], [27]]. In this study, we unveiled that FBXO2, encoded by a transcriptional target gene of p53, promotes the degradation of FABP5 via the CMA pathway. The degradation of FABP5 results in a decrease in PUFA levels, consequently leading to resistance to ferroptosis in CRC. Notably, our results revealed that the lack of PUFAs serves as a key determinant impairing the pro-ferroptotic function of p53, while AA supplementation cooperates with p53-inducing agents to suppress the growth of CRC by triggering ferroptosis (Fig. 7M).

Through transcriptomic analyses of HCT116 ^p53+/+^ and CAL51 cells treated with Nutlin-3 or chemotherapeutic agents, we identified that the expression FBXO2 is upregulated in response to the activation of p53, which is in line with a previous high-throughput study [42]. Importantly, our study demonstrated for the first time that *FBXO2* is an authentic p53-inducible target gene. First, utilizing the p53MH algorithm [32], we found a potential p53-RE upstream of the TIS of the *FBXO2* gene (Fig. 1M). Then, we conducted the luciferase reporter assay to confirm that the overexpression of p53 induces the luciferase activity driven by a promoter region containing the p53-RE from *FBXO2* (Fig. 1N). Furthermore, p53 can associate with the promoter region surrounding the p53-RE, with the p21 (*CDKN1A*) promoter serving as a positive control (Fig. 1O). Therefore, these findings uncover that *FBXO2* is a transcriptional target gene of p53.

Our results revealed that FBXO2 exerts tumor-promoting functions by enhancing the growth and migration of CRC cells (Fig. 2 and S2). This aligns with previous studies showing that elevated FBXO2 expression promotes tumor progression, facilitates p53 degradation, or serves as a prognostic indicator for unfavorable clinical outcomes in diverse human cancers [[43], [44], [45], [46], [47]]. The finding in our study is of particular interest because the tumor suppressor p53 activates a pro-tumorigenic protein. The activation of p53 elicits a potent cytotoxic impact on cancer cells. As a consequence, cancer cells may evolve adaptive responses under this stress condition. This could be achieved through the upregulation of various target genes that improve the viability of cancer cells, induce drug resistance, or provide feedback inhibition of p53, thereby sustaining their own survival and proliferation [30,[48], [49], [50], [51]]. Our study demonstrates that p53 activation of FBXO2 can partially alleviate the survival stress of cancer cells under oxidative stress caused by Erastin or RSL3 treatment (Fig. 6E and G, S6A-S6J), further highlighting the complex role of p53 in orchestrating cellular responses to various stress conditions.

The role of p53 in modulating ferroptosis sensitivity in CRC remains controversial. It has been reported that p53 limits ferroptosis by repressing the enzymatic activity of DPP4 via a transcription-independent manner in CRC cells, but not in breast cancer or osteosarcoma cells [25]. However, several other studies have shown that the inhibition of p53 by RFNG [26] or the long noncoding RNA HMG [27] leads to the upregulation of SLC7A11 and VKORC1L1 and thus the resistance to ferroptosis, thus suggesting that p53 may enhance ferroptosis sensitivity in CRC. Our study indicates that FABP5-mediated alteration of PUFA levels has a crucial impact on determining the role of p53 in regulating ferroptosis in CRC. While fatty acid-binding proteins (FABPs) are generally recognized for their roles in lipid transport, storage, and metabolism, our study reveals that FABP5 governs ferroptosis sensitivity by maintaining intracellular levels of PUFAs, such as AA. The degradation of FABP5 via p53-induced FBXO2 disrupts PUFA homeostasis, thereby impairing ferroptosis sensitivity in CRC (Fig. 3, Fig. 4, Fig. 5, Fig. 6). Therefore, our findings suggest that the role of p53 in restricting PUFA levels emerges as a predominant determinant of cell fate during ferroptosis in CRC, overshadowing its pro-ferroptotic function via the inhibition of antioxidant pathways [8,14,26,27] (Fig. 7M).

It is noteworthy that AA supplementation not only overcomes p53-mediated resistance to Erastin- or RSL3-induced ferroptosis but also collaborates with p53 to induce ferroptosis without the need for additional ROS stress (Fig. 7, S7, S8). Although p53 can promote lipid peroxidation in CRC by repressing the expression of antioxidant genes (e.g., *SLC7A11*, *VKORC1L1*) [8,14,26,27], limited intracellular PUFA availability constrains p53-induced lipid peroxidation, thereby blocking the onset of membrane rupture and subsequent ferroptosis. Nevertheless, it remains unknown how cellular contexts or stress conditions balance the dual functions of p53 – antioxidant defense versus PUFA restriction – to determine the divergent outcomes of ferroptosis in CRC.

In conclusion, our study as presented here uncovers the role of the FBXO2-FABP5 axis in p53-mediated ferroptosis resistance in CRC. p53 activated FBXO2 promotes the lysosomal degradation of FABP5 through the CMA pathway. The degradation of FABP5 leads to a decrease in the levels of PUFAs, consequently conferring resistance to ferroptosis in CRC. Crucially, AA supplementation counteracts p53-mediated ferroptosis resistance and, in the absence of additional ROS stress, collaborates with p53-inducing agents to drive ferroptosis.

## 4 Materials and methods

### 4.1 Colorectal cancer specimens

A total of 110 paraffin-embedded tissue sections obtained from the First Affiliated Hospital of Nanchang University were analyzed. Among them, 20 pairs of colorectal cancer tissues and matched normal tissues were used to analyze the expression of FBXO2 and FABP5, while 90 colorectal cancer tissues were used to analyze the overall survival based on the IHC score. The associations FBXO2 and FABP5 expression with overall survival are described in Table S1. This study was approved by the Human Research Ethics Committee of the First Affiliated Hospital of Nanchang University [(2023)CDYFYYLK(07–014)].

### 4.2 Mouse xenograft study

Mouse xenograft experiments were performed following ethical guidelines and were approved by the Animal Welfare Committee of Fudan University Shanghai Cancer Center (FUSCC-IACUC-2024012 and FUSCC-IACUC-2024133). Four-week-old female BALB/c nude mice were purchased from and maintained in the Laboratory Animal Science facility of Fudan University Shanghai Cancer Center. To assess the function of FBXO2 in vivo, 5 × 10^6^ HCT116 cells stably expressing an empty vector or the FBXO2 plasmid and 5 × 10^6^ HCT116 cells stably expressing shNC or shFBXO2 were subcutaneously implanted into nude mice. Tumor growth was monitored with an electronic digital caliper three times a week. For drug treatment, 5 × 10^6^ HCT116 cells were subcutaneously implanted into nude mice. Seven days later, the mice were randomly divided into four groups: control, 5-FU, AA and 5-FU + AA. 5-FU (10 mg/kg) and AA (4 mg/kg) were administrated via intraperitoneal injection (every other day for two weeks). The control group was administered with equal saline. Mice were monitored and weighed daily. The tumor volume was measured and calculated using the formula: volume = length × width^2^ × 0.52.

### 4.3 >Cell culture and transient transfection

Wild-type p53-harboring cancer cell lines, HCT116 ^p53+/+^ (RRID: CVCL_0291), RKO (RRID: CVCL_0504), CAL51 (RRID: CVCL_1110), MCF-7 (RRID: CVCL_0031), and A549 (RRID: CVCL_0023), mutant p53-harboring cancer cell lines, HT-29 (RRID: CVCL_0023), TOV112D (RRID: CVCL_3612) and OVCA420 (RRID: CVCL_3935), and p53-null cancer cell lines, HCT116 ^p53−/−^ (gift from Hua Lu lab [52]) and H1299 (RRID: CVCL_0060), human embryonic kidney cell line HEK293T (RRID: CVCL_0045) were cultured in DMEM containing 10 % fetal bovine serum, penicillin (100 U/mL), and streptomycin (0.1 mg/mL). All cells were incubated in a humidified environment containing 5 % CO_2_ at 37 °C. Cells were mycoplasma-free and authenticated by PCR analysis. Plasmids and siRNAs were transiently transfected using Hieff Trans Liposomal transfection reagent according to the manufacturer's protocol (Yeasen, Shanghai, China).

### 4.4 Plasmids, antibodies, and reagents

Flag-tagged pENTER-FBXO2 plasmids were purchased from Vigene Biosciences (Shandong, China). Human FBXO2, FABP5, HSPA8, LAMP2A and CULIN1 were cloned into the vector Myc-pcDNA, Flag-pcDNA, PCDH and PCMV-HA. For the luciferase reporter assay, sequences upstream of the transcription initiation site of FBXO2, as indicated in the figure, were cloned into the vector pGL3-basic. The KOD-Plus-Mutagenesis Kit (TOYOBO, Osaka, Japan) was used to make the Flag-FABP5-Mut and Flag-FBXO2 (ΔF-box) construct. Primers used for plasmid construction are listed in Table S2. The anti-Flag antibody (F1804) was purchased from Sigma-Aldrich (St. Louis, MO, USA). The anti-FBXO2 (14590-1-AP), anti-FABP5 (12348-1-AP), anti-Myc (60003-2-Ig and 16286-1-AP), anti-GAPDH (60004-1-Ig), anti-α-Tubulin (66031-1-Ig), anti-β-Actin (60008-1-Ig) anti-HSPA8 (10654-1-AP) and anti-Vinculin (66305-1-Ig) antibodies, the secondary antibodies for rabbit (ARG65351) and mouse (ARG65350) were purchased from Proteintech (Wuhan, Hubei, China). The anti-FBXO2 (sc-393873), anti-p53 (sc-126) antibodies, and the normal mouse IgG (sc-2025) were purchased from Santa Cruz Biotechnology (Santa Cruz, CA, USA). The anti-p21 (#2947), anti-SLC7A11 (#98051), anti-PUMA (#4976), anti-HA (#3724) antibody and the normal rabbit IgG (#2729) were purchased from Cell Signaling Technology (Danvers, MA, USA). The anti-p53 (Ab179477) and anti-ALOX15 (Ab244205) antibodies were purchased from Abcam (Cambridge, MA, USA). The anti-GPX4 (A1933) antibody was purchased from ABclonal (Wuhan, Hubei, China). Nutlin-3, Cisplatin, 5-fluorouracil (5-FU), actinomycin D (Act.D), Etoposide, Alrizomadlin (APG-115), Olaparib, MG132, Liproxstatin-1 (Lipro-1), Ferrostatin-1 (Ferro-1), RSL3, Z-VAD-FMK, Chloroquine (CQ), and Oxaliplatin (Oxa) were purchased from MedChemExpress (Shanghai, China), Erastin and Arachidonic acid (AA) were purchased from Selleck (Shanghai, China). Proteins were visualized with the ECL chemiluminescence reagent (Yeasen).

### 4.5 Reverse transcription and quantitative RT-PCR analysis

Total RNAs were extracted from cells using RNAiso Plus following the manufacturer's protocol (Takara, Dalian, China). The PrimeScript RT kit with gDNA Eraser (Vazyme, Nanjing, China) was used for reverse transcription. Quantitative RT-PCR (qRT-PCR) was conducted using SYBR Green Premix according to the manufacturer's protocol (Vazyme, Nanjing, China). The primers for RT-qPCR used in this study are listed in Table S3.

### 4.6 RNA interference and generation of stable cell lines

All siRNAs were synthesized and purified by GenePharma (Shanghai, China). The cells were seeded in 6-well plates at an appropriate density. SiRNAs were transfected using Hieff Trans Liposomal transfection reagent following the manufacturer's protocol (Yeasen, Shanghai, China). After being cultured for 24–48 h, cells were harvested for RT-qPCR or IB analyses. To establish stable knockdown cell lines, the shRNA plasmid was co-transfected with the packaging plasmids, pSPAX2 and PMD2.0G, into HEK293T cells using polyethyleneimine (PEI) (Sigma-Aldrich, St. Louis, MO, USA). The lentivirus particles were collected 48 h after transfection and then used for infection of HCT116 and RKO cells. Stable cells were selected with puromycin (Yeasen) and verified by IB. The sequences of siRNAs and shRNAs used in this study are listed in Table S4 and Table S5, respectively.

### 4.7 Luciferase reporter assay

The FBXO2 promoter region −1719 bp to −1690 bp was amplified from the genomic DNA of HCT116 ^p53+/+^ cells and cloned into the pGL3-basic vector. Cells were seeded in 24-well plates and co-transfected with the plasmids encoding p53, p53-RE-WT and Renilla luciferase. Luciferase activity was measured 48 h post transfection according to the manufacturer's instructions using a dual-luciferase reporter system (Promega, Madison, WI, USA).

### 4.8 Immunoblotting and immunoprecipitation assays

Cells were harvested and lysed in lysis buffer [50 mM Tris/HCl (pH7.5), 0.5 % NP-40 (Nonidet P-40), 1 mM ethylene diamine tetraacetic acid (EDTA), 150 mM NaCl, 1 mM dithiothreitol, 0.2 mM phenylmethylsulfonyl fluoride, 10 mM pepstatin A, and 1 mM leupeptin] or RIPA buffer [50 mM Tris-HCl (pH 8.0), 5 mM EDTA, 1 % NP-40, 0.5 % deoxycholate, 0.1 % SDS, 150 mM NaCl, and protease inhibitor cocktail], and equal amounts of clear cell lysate were used for immunoblotting (IB) analysis. Original blots can be found as Supplemental Material. Proteins of 0.5–1 mg were incubated with the indicated antibodies at 4 °C for 4–6 h or overnight. Protein A or G beads (Santa Cruz Biotechnology) were then added into the mixture for additional 1–2 h at 4 °C. The beads were washed at least five times with lysis buffer. Bound proteins were detected by IB with antibodies as indicated in the figures.

### 4.9 Chromatin immunoprecipitation assay

ChIP was conducted according to the procedure as previously described [53]. Cells were crosslinked with 37 % formaldehyde for 10 min at room temperature and neutralized with glycine to a final concentration of 0.2 mol/L for 5 min. Cells were harvested after being washed three times with cold PBS, suspended in cell lysis buffer, and incubated on ice for 30 min. Nuclei were resuspended in 0.5 mL nuclear lysis buffer (50 mmol/L Tris-HCl pH 8.0, 10 mmol/L EDTA, 1 % SDS, and proteinase inhibitor cocktail). After sonication (30 cycles with 30 s on and 30 s off), lysates were centrifuged at 12,000×*g* for 5 min, and the supernatants were mixed with anti-p53 or IgG for overnight and then incubated with protein A/G beads for 2 h at 4 °C. Beads were washed sequentially with Low Salt Wash Buffer (50 mmol/L Tris-HCl pH 8.0, 0.1 % SDS, 0.5 % deoxycholate, 1 mmol/L EDTA, 1 % NP-40, and 150 mmol/L NaCl), High Salt Wash Buffer (50 mmol/L Tris-HCl pH 8.0, 0.1 % SDS, 0.5 % deoxycholate,1 mmol/L EDTA, 1 % NP-40, and 500 mmol/L NaCl), LiCl Wash Buffer (50 mmol/L Tris-HCl pH 8.0, 250 mmol/L LiCl, 0.1 % SDS, 0.5 % deoxycholate, 1 mmol/L EDTA, and 1 % NP-40), and TE Buffer (10 mmol/L Tris-HCl pH 8.0, and 1 mmol/L EDTA). The protein-DNA complexes were eluted with ChIP elution buffer (1 % SDS and 0.1 mol/L NaHCO3). After decrosslinking for 2 h at 62 °C, DNA was extracted and analyzed by qPCR. The primers for ChIP-qPCR are listed in Table S3.

### 4.10 Mass spectrometry analysis

RKO cells stably expressing Flag-FBXO2 or the control vector were subjected to the co-IP assay using the anti-Flag antibody. Bound proteins were analyzed by Coomassie staining and the specific bands were collected for mass spectrometry analysis (PTM BioLab, Hangzhou, China).

### 4.11 Cell viability assay

The Cell Counting Kit-8 (CCK-8) (Yeason, Shanghai, China) was used for the cell viability assay according to the manufacturer's instructions. Cell suspensions were plated at a density of 2000–4000 cells per well on 96-well culture plates, 6–12 h after transfection. 10 % WST-8 was added to each well every 24 h, and the absorbance of the samples was measured at 450 nm.

### 4.12 Flow cytometric assay of apoptosis

Apoptosis was examined by flow cytometry using the Annexin-V-PE/7-AAD kit (Vazyme) according to the manufacturer's protocol. Briefly, cells were harvested at 48 h after being transfected with the indicated siRNAs or plasmids and stained with Annexin-V-PE and 7-AAD for 20 min at room temperature in the dark. The percentages of apoptotic cells were measured by flow cytometry (Beckman Coulter).

### 4.13 Colony formation assay

After 4–6 h transfection, the cell suspension was seeded into a 6-well culture plate at a density of 500–1000 cells per well. Cells were cultured for about two weeks and the medium was changed every 3 days. Cells were then fixed with methanol and stained with crystal violet solution for 2 h or overnight. Colonies were counted using the ImageJ software.

### 4.14 Transwell migration assay

The migration assay was conducted in a 24-well plate with a transwell chamber insert. The upper chamber was filled with 0.5-1 × 10^5^ cells suspended in 200 μL of serum-free medium. The lower chamber was filled with complete medium. After being cultured for 36–72 h at 37 °C, the cells on the upper surface were washed away, while the cells on the lower layer were fixed with methanol and stained with crystal violet (BBI Life Sciences) overnight. The migratory cells were photographed. ImageJ was used to calculate the number of migratory cells.

### 4.15 Immunofluorescence staining and confocal assays

Cells were seeded on the glass slide in 6-well plates and were fixed with methanol at −20 °C overnight. The fixed cells were washed by PBS and blocked with 8 % bovine serum albumin (BSA) in PBS for 1 h followed by incubation with primary antibodies at 4 °C overnight. The cells were then washed and incubated with the corresponding secondary fluorescent antibodies. Cells were then placed on slides and stained with DAPI solution (Sigma-Aldrich, USA). Images were taken with a Leica fluorescence confocal microscope.

### 4.16 Immunohistochemistry (IHC)

The paraffin-embedded sections were deparaffinized and rehydrated, underwent antigen retrieval and then incubated with anti-FBXO2 antibody or the anti-FABP5 antibody at 4 °C overnight. After extensive washing with PBS, the sections were incubated with HRP-conjugated secondary antibody for 1 h, developed in DAB reagents, counterstained with hematoxylin, dehydrated with a graded alcohol series, and mounted with coverslips and mounting medium. Interpretation of the IHC results was performed by two independent pathologists.

### 4.17 Untargeted lipidomic analysis

The experiments and data analysis were supported by Shanghai Applied Protein Technology Co., Ltd. Reagents including MS-grade methanol. Lipids were extracted according to MTBE method. Briefly, a 200 μL volume of water was added to 30 mg sample and vortexed for 5 s. Subsequently, 240 μL of pre-cooled methanol was added and the mixture vortexed for 30 s. After that, 800 μL of MTBE was added and the mixture was ultrasound 20 min at 4 °C followed by sitting still for 30 min at room temperature. The solution was centrifuged at 14000 g for 15 min at 10 °C and the upper organic solvent layer was obtained and dried under nitrogen. Reverse phase chromatography was selected for LC separation using CSH C18 column (1.7 μm, 2.1 mm × 100 mm, Waters). The lipid extracts were re-dissolved in 200 μL 90 % isopropanol/acetonitrile, centrifuged at 14000 g for 15 min, finally 3 μL of sample was injected. Solvent A was acetonitrile–water (6:4, v/v) with 0.1 % formic acid and 0.1 mM ammonium formate and solvent B was acetonitrile–isopropanol (1:9, v/v) with 0.1 % formic acid and 0.1 mM ammonium formate. The initial mobile phase was 40 % solvent B at a flow rate of 300 μL/min. It was held for 3.5 min, and then linearly increased to 75 % solvent B in 9.5 min, and then linearly increased to 99 % solvent B in 6 min, followed by equilibrating at 40 % solvent B for 5 min. Mass spectra were acquired by Q-Exactive Plus in positive and negative mode, respectively. ESI parameters were optimized and preset for all measurements as follows: Source temperature, 300 °C; Capillary Temp, 350 °C, the ion spray voltage was set at 3000 V, S-Lens RF Level was set at 50 % and the scan range of the instruments was set at *m*/*z* 200–1800. “Lipid Search” is a search engine for the identification of lipid species based on MS/MS math. Lipid Search contains more than 30 lipid classes and more than 1,500,000 fragment ions in the database. Both mass tolerance for precursor and fragment were set to 5 ppm.

### 4.18 BODIPY™ 581/591C11 assay

BODIPY™ 581/591C11 dye (Invitrogen, Shanghai, China) was used to detect ROS in cells and membranes. Cells were seeded in six-well plates at appropriate density and treated with the indicated agents for 24–48 h. Cells in each well were then incubated with fresh FBS-free medium containing 5 μM BODIPY™ 581/591C11 dye at 37 °C for 30 min in the dark. Cells were washed twice and resuspended in PBS. The fluorescence intensity of cells labelled with BODIPY™ 581/591C11 was analyzed by flow cytometry (CytoFLEX S, Beckman Coulter, USA). Fluorescence intensity of living cells was recorded using gate technique. Oxidation of the polyunsaturated butadienyl portion of the dye results in a shift of the fluorescence emission peak from ∼590 nm (FL2) to ∼510 nm (FL1). Each set of data was obtained from three independent replicates and analyzed by FlowJo 10.8.1 version.

### 4.19 Malondialdehyde assay

Intracellular Malondialdehyde (MDA) levels were determined by the Malondialdehyde (MDA) assay kit (DOJINDO, Shanghai, China) according to the manufacturer's protocol. Cells were treated with the indicated agents for 24–48 h, then harvested in antioxidant PBS solution and suspended in lysis buffer and working solution. The mixture was incubated for 15 min at 95 °C followed by ice-cooling for 5 min. The supernatant was collected by centrifugation at 10000*g*, 4 min, and the fluorescence intensity was measured by a microplate reader at ex 540 nm and em 590 nm.

### 4.20 GSH assay

The intracellular levels of GSH were measured using GSH and GSSG Assay Kit (Beyotime). According to the manufacturer's instructions, the IsoA-treated cells were collected and homogenized. Briefly, cells were washed with 1 × PBS and collected, resuspended in three times the volume of protein removal reagent M solution. Cell samples were subjected to two rapid freeze–thaw cycles using liquid nitrogen and 37 °C water bath, and then centrifuged at 10000 g for 10 min at 4 °C. Corresponding detection reagents were added to an appropriate amount of cell supernatant. After 25 min, GSH was detected by a microplate analyzer at an absorbance of 412 nm. Then GSH content was calculated according to the standard curve.

### 4.21 Statistical analysis

All in vitro data were obtained from three independent replicate experiments. Data were shown as means ± standard deviation (SD). Student's *t*-test or one-way ANOVA was used to compare the differences between two or more groups. The overall survival of patients with colorectal cancer was analyzed using the Kaplan-Meier method. p < 0.05 was considered statistically significant. ns represent statistically non-significant, ∗p < 0.05, ∗∗p < 0.01, ∗∗∗p < 0.001.

## Availability of data and materials

The data generated or analyzed during this study are included in this published article and supplementary materials.

## Funding

This work was supported by the 10.13039/501100001809National Natural Science Foundation of China (82173022 to Q.H., 82273098 and 82472714 to X.Z., 82172891 to T.H., 82260491 to J.D., and 82403120 to Y.G.), the 10.13039/100020732Innovative Research Team of High-level Local University in Shanghai, 10.13039/100019767International Science and Technology Cooperation Projects in Henan Province Projects in Henan Province (252102521001), the Key Projects of Jiangxi Province (20232ACB206044), and the252102521001 “Thousand Talents Program” for introducing and cultivating high-level talents in innovation and entrepreneurship in Jiangxi Province (jxsq2023201108).

## CRediT authorship contribution statement

**Jing Tong:** Data curation, Formal analysis, Investigation, Methodology, Validation, Writing – original draft. **Tao Han:** Data curation, Formal analysis, Funding acquisition, Investigation, Methodology, Writing – original draft. **Jun Deng:** Conceptualization, Formal analysis, Funding acquisition, Investigation, Methodology, Supervision. **Yu Gan:** Conceptualization, Funding acquisition, Investigation, Methodology. **Ruiwen Ruan:** Formal analysis, Investigation, Methodology. **Wei Zhao:** Investigation, Methodology. **Chen Xiong:** Investigation, Methodology. **Quan Liao:** Investigation, Methodology. **Shiqi Chen:** Investigation. **Huitong Bu:** Investigation. **Jianping Xiong:** Investigation, Resources, Supervision. **Xiang Zhou:** Conceptualization, Formal analysis, Funding acquisition, Investigation, Project administration, Resources, Supervision, Writing – original draft, Writing – review & editing. **Qian Hao:** Conceptualization, Formal analysis, Funding acquisition, Investigation, Methodology, Project administration, Resources, Supervision, Writing – original draft, Writing – review & editing.

## Declaration of competing interest

The authors declare no competing interests.

## Data Availability

Data will be made available on request.
