# Supplementary material for: p53 and fatty acids collaborate to trigger ferroptosis via the FBXO2-FABP5 axis in colorectal cancer
Source: Redox Biol. 2026 Jan 19;90:104043. doi: 10.1016/j.redox.2026.104043 (PMC12865648; doi:10.1016/j.redox.2026.104043)
Supplement: Multimedia component 1 [file mmc1.pdf]

Supplementary Information

p53 and fatty acids collaborate to trigger ferroptosis via the FBXO2-FABP5 axis in colorectal cancer

Jing Tong[1,2,#], Tao Han[3,#], Jun Deng[4,5,#], Yu Gan[1,2], Ruiwen Ruan[4,5], Wei Zhao[1,2], Chen Xiong[1,2], Quan Liao[1,2], Shiqi Chen[1,2], Huitong Bu[3], Jianping Xiong[4,5], Xiang Zhou[1,2,6,*], and Qian Hao[1,2,*]

[1] Cancer Institute, Fudan University Shanghai Cancer Center, Shanghai 200032, China

[2] Department of Oncology, Shanghai Medical College, Fudan University, Shanghai 200032, China

[3] Xinxiang Key laboratory for Molecular Oncology, Institutes of Health Central Plains, Xinxiang Medical University, Xinxiang 453003, China

[4] Department of Oncology, The First Affiliated Hospital, Jiangxi Medical College, Nanchang University, Nanchang 330006, Jiangxi, China

[5] Jiangxi Key Laboratory for Individual Cancer Therapy, Nanchang 330006, Jiangxi, China

[6] Department of Breast Surgery, Key Laboratory of Breast Cancer in Shanghai, Fudan University Shanghai Cancer Center, Shanghai 200032, China

[#] Equal contribution

[*] Correspondence:

Xiang Zhou, Email: xiangzhou@fudan.edu.cn

Qian Hao, Email: haoqian@fudan.edu.cn

Running title: p53 and PUFAs coordinately trigger ferroptosis

Supplementary figure legends

Figure S1. FBXO2 is induced by p53, Related to Figure 1.

(A–C) Transcriptomic analyses reveals FBXO2 as a potential target gene of p53.

(D–F) qPCR analysis of the expression of FBXO2 mRNA in CAL51 (D), MCF-7 (E), and A549 (F) cells treated with or without the indicated agents for 24 h.

(G and H) qPCR analysis of FBXO2 mRNA expression in CAL51 (G) and MCF-7 (H) cells treated with the siRNAs and agents as indicated.

(I–L) qPCR analysis of FBXO2 mRNA expression in p53-null HCT116 $^{p53-/-}$ (I) and H1299 (J) cells and p53-mutated TOV112D (K) and OVCA420 (L) cells, treated with or without the indicated agents for 24 h.

Data are represented as mean ± SD, $n = 3$. * $p < 0.05$, *** $p < 0.001$, ns, not significant.

Figure S2. Ablation of FBXO2 inhibits the growth and progression of colorectal cancer, Related to Figure 2.

(A–H) HCT116 $^{p53+/+}$ and RKO cells were transfected with control or FBXO2 siRNAs, followed by the cell viability assay (A and B), colony formation assay (C and D), flow cytometric assay (E and F), and transwell migration assay (G and H).

Data are represented as mean ± SD, $n = 3$. ** $p < 0.01$, *** $p < 0.001$. Scale bars, 100 μm.

(I–K) The growth rate (I), weight (J), and size (K) of xenograft tumors derived from HCT116 $^{p53+/+}$ cells stably expressing shNC or shFBXO2.

Data in (I) and (J) are represented as mean ± SD, $n = 6$. * $p < 0.05$, *** $p < 0.001$.

Figure S3. p53 inhibits the level of FABP5, Related to Figure 3.

(A) HEK293T cells were transfected with the indicated plasmids, followed by IF staining and microscopy analysis.

(B and C) HCT116 $^{p53+/+}$ (B) and RKO (C) cells were treated with different doses of Nutlin-3, followed by IB analysis.

(D and E) HCT116 $^{p53+/+}$ (D) and RKO (E) cells were treated with different doses of APG-115, followed by IB analysis.

(F and G) HCT116 $^{p53+/+}$ (F) and RKO (G) cells were treated with different doses of 5-FU, followed by IB analysis.

(H and I) HCT116 $^{p53-/-}$ (H) and H1299 (I) cells were transfected with an empty vector or pcDNA3.1-p53, followed by IB analysis.

(J) HCT116 $^{p53-/-}$ cells were treated with different doses of Nutlin-3, followed by IB analysis.

(K) The negative correlation between the expression of FBXO2 and FABP5 based on IHC scores in 20 CRC samples.

(L) FBXO2 and FABP5 are prognostic factors in CRC.

Figure S4. Clinical significance of FBXO2 and FABP5 in colorectal cancer, Related to Figure 3.

(A) The FBXO2 mRNA level is higher in colon adenocarcinoma (COAD) compared to normal tissues.

(B) The protein level of FABP5 is lower in colon cancer compared to normal tissues.

(C) Higher levels of FBXO2 are associated with worse recurrence-free survival in colon cancer.

(D) Higher levels of FABP5 are associated with better recurrence-free survival in colon cancer.

Figure S5. FABP5 enhances ferroptosis sensitivity in colorectal cancer cells, Related to Figure 5.

(A and B) HCT116 $^{p53+/+}$ (A) and RKO (B) cells were transfected with the indicated plasmids and treated with different doses of Erastin for 24 h, followed by the cell viability assay.

(C and D) HCT116 $^{p53+/+}$ (C) and RKO (D) cells stably expressing shNC or shFABP5 were exposed to different doses of RSL3 for 24 h, followed by the cell viability assay.

(E and F) HCT116 $^{p53+/+}$ (E) and RKO (F) cells were transfected with the indicated plasmids and treated with or without Erastin for 24 h, followed by the MDA assay.

(G and H) HCT116 $^{p53+/+}$ (G) and RKO (H) cells were transfected with the indicated plasmids and treated with or without Erastin for 24 h, followed by the GSH assay.

Data are represented as mean ± SD, $n = 3$. * $p < 0.05$, ** $p < 0.01$, *** $p < 0.001$.

Figure S6. FBXO2 promotes ferroptosis resistance in colorectal cancer cells, Related to Figure 6.

(A and B) HCT116 $^{p53+/+}$ (A) and RKO (B) cells were transfected with the indicated plasmids and treated with different doses of Erastin for 24 h, followed by the cell viability assay.

(C and D) HCT116 $^{p53+/+}$ (C) and RKO (D) cells were transfected with the indicated plasmids and treated with different doses of RSL3 for 24 h, followed by the cell viability assay.

(E and F) HCT116 $^{p53+/+}$ (E) and RKO (F) cells were transfected with the indicated plasmids and treated with or without Erastin for 24 h, followed by the MDA assay.

(G and H) HCT116 $^{p53+/+}$ (G) and RKO (H) cells were transfected with the indicated shRNAs and treated with different doses of Erastin for 24 h, followed by the cell viability assay.

(I and J) HCT116 $^{p53+/+}$ (I) and RKO (J) cells were transfected with the indicated shRNAs and treated with or without Erastin for 24 h, followed by the MDA assay.

(K and L) HCT116$^{p53+/+}$ (K) and RKO (L) cells were treated with DMSO, Nutlin-3, and RSL3 as indicated for 48 h, followed by the cell viability assay.

Data are represented as mean ± SD, $n = 3$. * $p < 0.05$, ** $p < 0.01$, *** $p < 0.001$.

Figure S7. Arachidonic acid cooperates with p53-inducing agents to trigger ferroptosis, Related to Figure7.

(A and B) HCT116 $^{p53+/+}$ (A) and RKO (B) cells were treated with the indicated agents for 48

h, followed by qPCR analysis.

(C and D) HCT116 $^{p53+/+}$ (C) and RKO (D) cells were treated with the indicated agents for 48 h, followed by IB analysis.

(E and F) HCT116 $^{p53+/+}$ (E) and RKO (F) cells were treated with the indicated agents for 48 h, followed by the cell viability assay.

(G and H) HCT116 $^{p53+/+}$ (G) and RKO (H) cells were treated with the indicated agents for 48 h, followed by the BODIPY^{TM} 581/591 C11 assay.

(I and J) HCT116 $^{p53+/+}$ and RKO cells were treated with the indicated agents for 48 h, followed by the IB analysis.

(K and L) HCT116 $^{p53+/+}$ (K) and RKO (L) cells were treated with the indicated agents for 48 h, followed by the cell viability assay.

(M and N) HCT116 $^{p53+/+}$ (M) and RKO (N) cells were treated with the indicated agents for 48 h, followed by the BODIPYTM 581/591 C11 assay.

(O and P) HCT116 $^{p53+/+}$ (O) and RKO (P) cells were treated with the indicated agents for 48 h, followed by the MDA assay.

Data are represented as mean ± SD, n = 3. ** p < 0.01, *** p < 0.001.

Figure S8. Arachidonic acid cooperates with p53-inducing agents to trigger ferroptosis, Related to Figure7.

(A and B) HCT116 $^{p53+/+}$ (A) and RKO (B) cells were treated with the indicated agents for 48 h, followed by the cell viability assay.

(C and D) HCT116 $^{p53+/+}$ (C) and RKO (D) cells were treated with the indicated agents for 48 h, followed by the BODIPY^{TM} 581/591 C11 assay.

Data are represented as mean ± SD, *n* = 3. ** $p < 0.01$, *** $p < 0.001$.

Figure S1

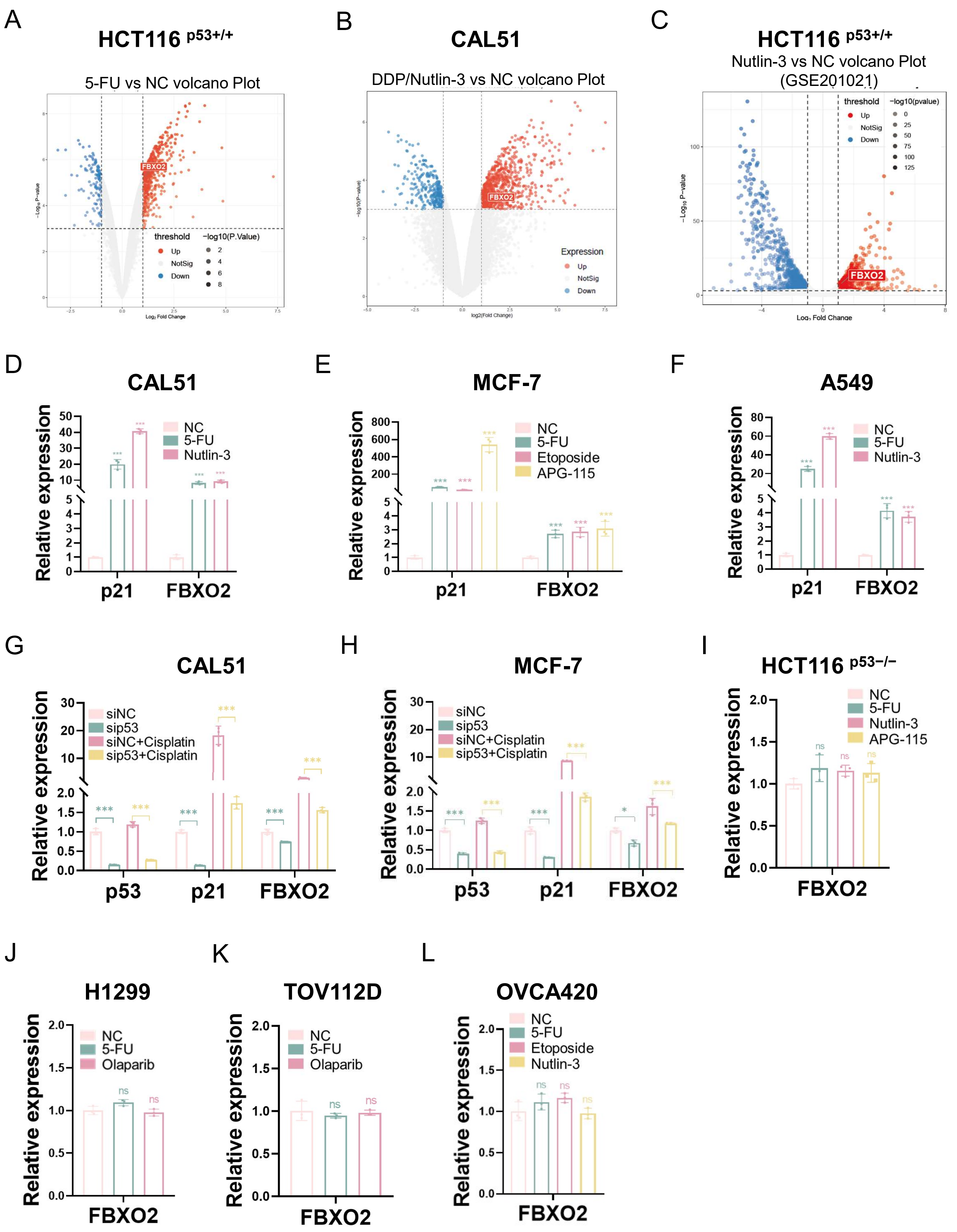

A
HCT116 p53+/+
5-FU vs NC volcano Plot
FBXO2
threshold
Up
NotSig
Down
−log10(P.Value)
B
CAL51
DDP/Nutlin-3 vs NC volcano Plot
FBXO2
Expression
Up
NotSig
Down
C
HCT116 p53+/+
Nutlin-3 vs NC volcano Plot
(GSE201021)
FBXO2
threshold
−log10(pvalue)
Log2 Fold Change
D
CAL51
NC
5-FU
Nutlin-3
Relative expression
p21
FBXO2
E
MCF-7
NC
5-FU
Etoposide
APG-115
Relative expression
p21
FBXO2
F
A549
NC
5-FU
Nutlin-3
Relative expression
p21
FBXO2
G
CAL51
siNC
sip53
siNC+Cisplatin
sip53+Cisplatin
Relative expression
p53
p21
FBXO2
H
MCF-7
siNC
sip53
siNC+Cisplatin
sip53+Cisplatin
Relative expression
p53
p21
FBXO2
I
HCT116 p53−/−
NC
5-FU
Nutlin-3
APG-115
Relative expression
FBXO2
J
H1299
NC
5-FU
Olaparib
Relative expression
FBXO2
K
TOV112D
NC
5-FU
Olaparib
Relative expression
FBXO2
L
OVCA420
NC
5-FU
Etoposide
Nutlin-3
Relative expression
FBXO2

Figure S2

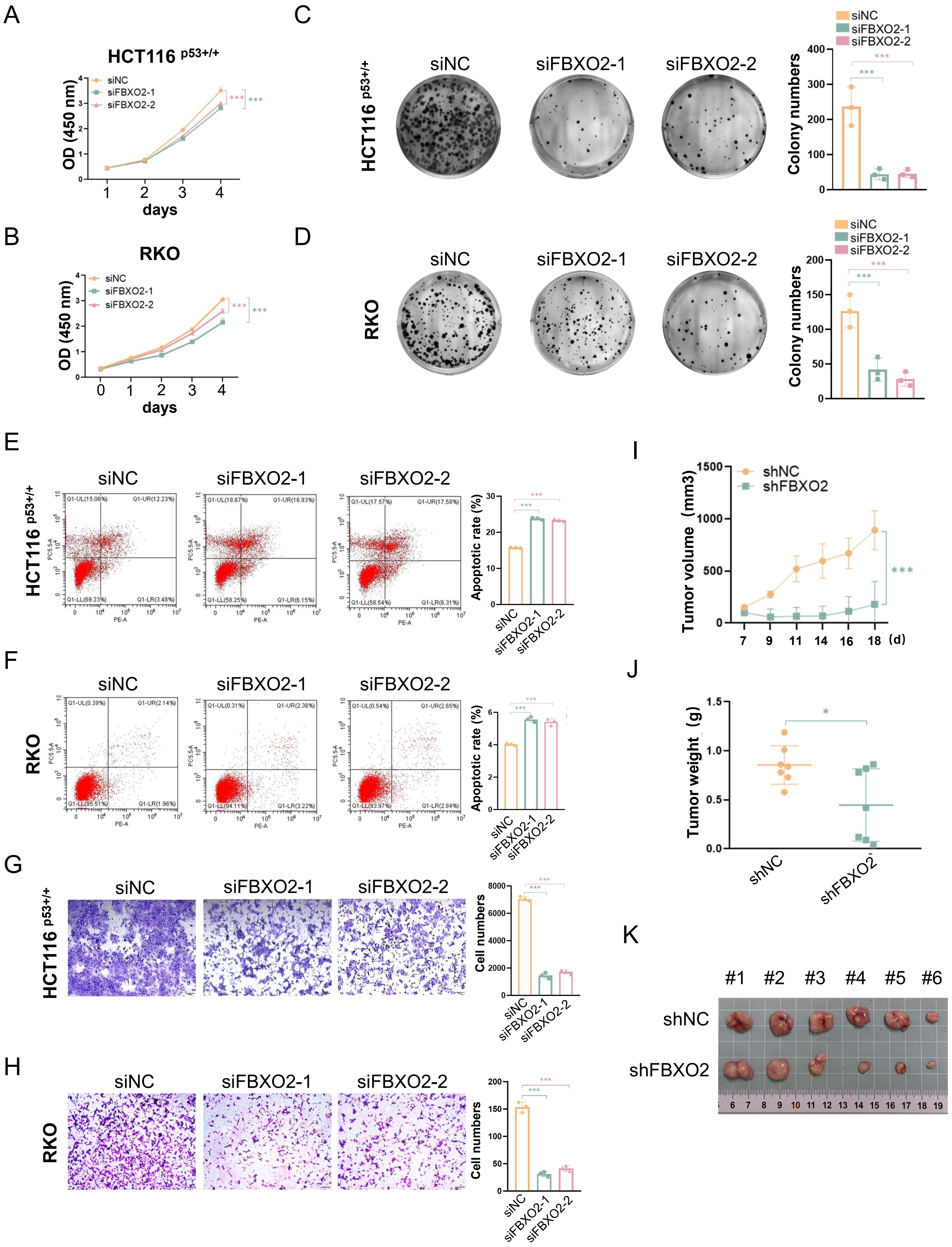

A
HCT116 p53+/+
siNC
siFBXO2-1
siFBXO2-2
OD (450 nm)
days
***
***
B
RKO
siNC
siFBXO2-1
siFBXO2-2
OD (450 nm)
days
C
HCT116 p53+/+
siNC
siFBXO2-1
siFBXO2-2
Colony numbers
D
RKO
siNC
siFBXO2-1
siFBXO2-2
Colony numbers
E
HCT116 p53+/+
siNC
siFBXO2-1
siFBXO2-2
Apoptotic rate (%)
F
RKO
siNC
siFBXO2-1
siFBXO2-2
Apoptotic rate (%)
G
HCT116 p53+/+
siNC
siFBXO2-1
siFBXO2-2
Cell numbers
H
RKO
siNC
siFBXO2-1
siFBXO2-2
Cell numbers
I
shNC
shFBXO2
Tumor volume (mm3)
(d)
***
J
Tumor weight (g)
shNC
shFBXO2
*
K
#1
#2
#3
#4
#5
#6
shNC
shFBXO2

Figure S3

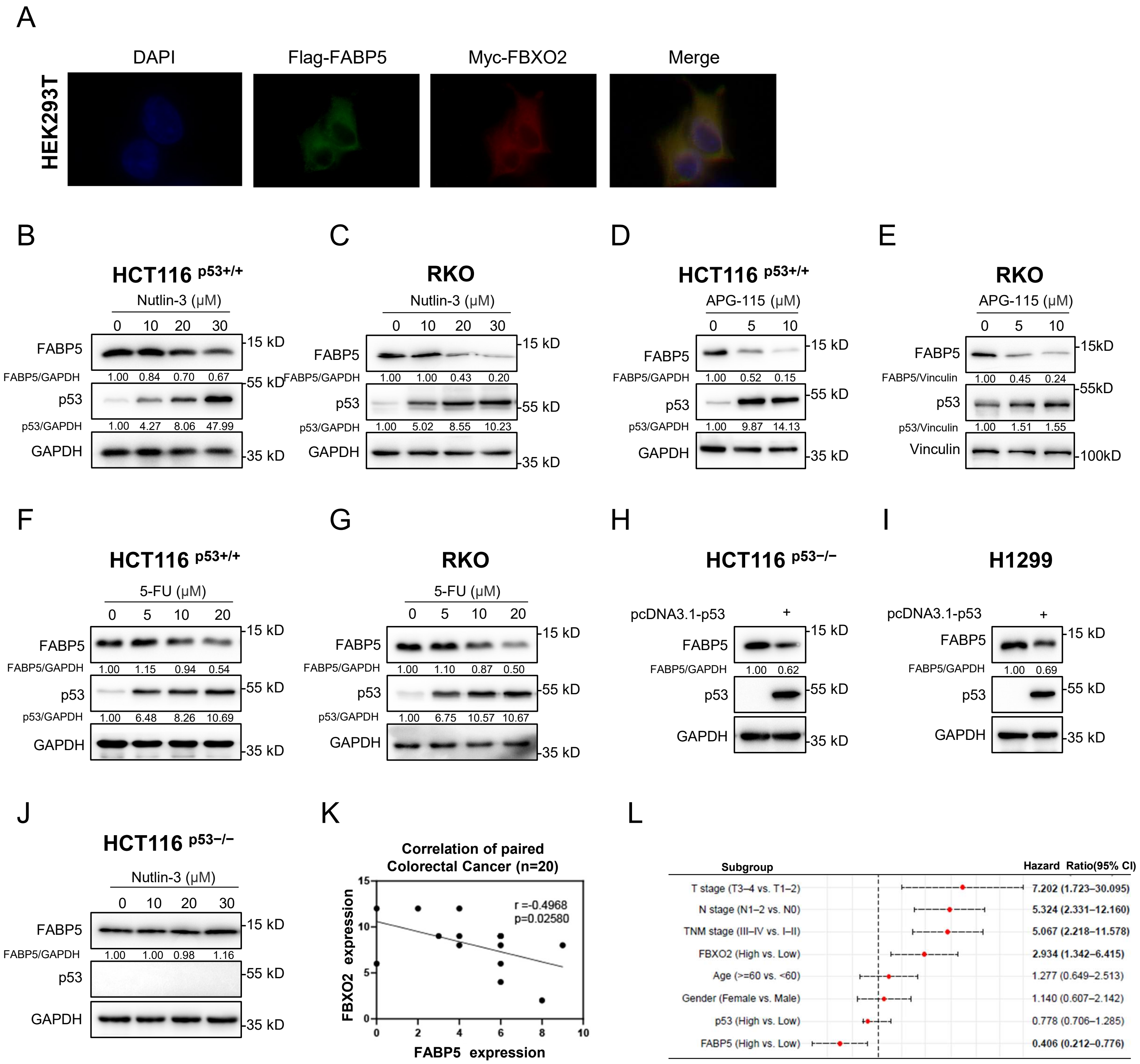

A
DAPI
Flag-FABP5
Myc-FBXO2
Merge
HEK293T
B
HCT116 p53+/+
Nutlin-3 (μM)
0 10 20 30
FABP5
FABP5/GAPDH 1.00 0.84 0.70 0.67
p53
p53/GAPDH 1.00 4.27 8.06 47.99
GAPDH
15 kD
55 kD
35 kD
C
RKO
Nutlin-3 (μM)
0 10 20 30
FABP5
FABP5/GAPDH 1.00 1.00 0.43 0.20
p53
p53/GAPDH 1.00 5.02 8.55 10.23
GAPDH
D
HCT116 p53+/+
APG-115 (μM)
0 5 10
FABP5/GAPDH 1.00 0.52 0.15
p53/GAPDH 1.00 9.87 14.13
E
RKO
APG-115 (μM)
0 5 10
FABP5/Vinculin 1.00 0.45 0.24
p53/Vinculin 1.00 1.51 1.55
Vinculin
15kD
55kD
100kD
F
HCT116 p53+/+
5-FU (μM)
0 5 10 20
FABP5/GAPDH 1.00 1.15 0.94 0.54
p53/GAPDH 1.00 6.48 8.26 10.69
G
RKO
5-FU (μM)
0 5 10 20
FABP5/GAPDH 1.00 1.10 0.87 0.50
p53/GAPDH 1.00 6.75 10.57 10.67
H
HCT116 p53−/−
pcDNA3.1-p53 +
FABP5/GAPDH 1.00 0.62
I
H1299
pcDNA3.1-p53 +
FABP5/GAPDH 1.00 0.69
J
HCT116 p53−/−
Nutlin-3 (μM)
0 10 20 30
FABP5/GAPDH 1.00 1.00 0.98 1.16
K
Correlation of paired
Colorectal Cancer (n=20)
r =-0.4968
p=0.02580
FBXO2 expression
FABP5 expression
L
Subgroup
Hazard Ratio(95% CI)
T stage (T3–4 vs. T1–2) 7.202 (1.723–30.095)
N stage (N1–2 vs. N0) 5.324 (2.331–12.160)
TNM stage (III–IV vs. I–II) 5.067 (2.218–11.578)
FBXO2 (High vs. Low) 2.934 (1.342–6.415)
Age (>=60 vs. <60) 1.277 (0.649–2.513)
Gender (Female vs. Male) 1.140 (0.607–2.142)
p53 (High vs. Low) 0.778 (0.706–1.285)
FABP5 (High vs. Low) 0.406 (0.212–0.776)

Figure S4

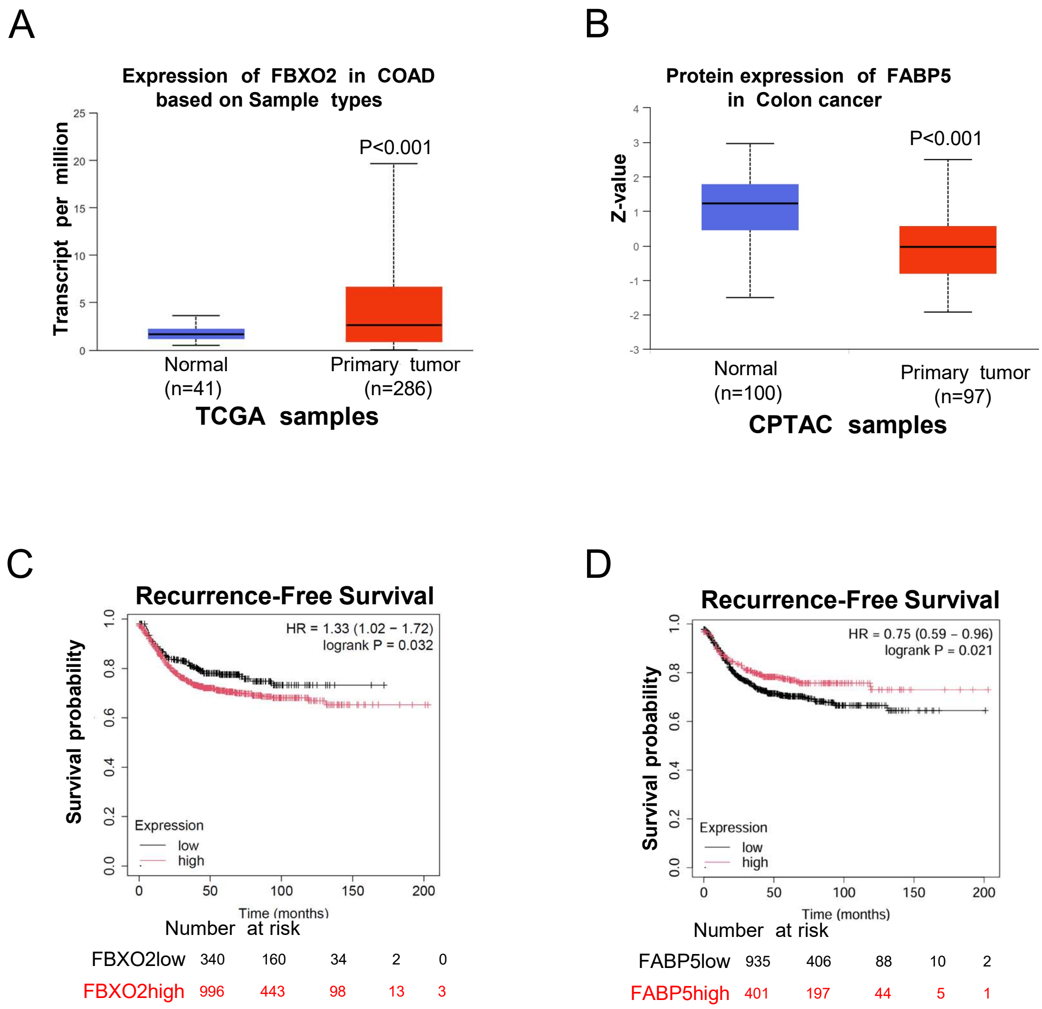

A
Expression of FBXO2 in COAD
based on Sample types
Transcript per million
25
20
15
10
5
0
P<0.001
Normal
(n=41)
Primary tumor
(n=286)
TCGA samples
B
Protein expression of FABP5
in Colon cancer
Z-value
4
3
2
1
0
-1
-2
-3
P<0.001
Normal
(n=100)
Primary tumor
(n=97)
CPTAC samples
C
Recurrence-Free Survival
HR = 1.33 (1.02 − 1.72)
logrank P = 0.032
Survival probability
1.0
0.8
0.6
0.4
0.2
0.0
Expression
low
high
0
50
100
150
200
Time (months)
Number at risk
FBXO2low 340 160 34 2 0
FBXO2high 996 443 98 13 3
D
Recurrence-Free Survival
HR = 0.75 (0.59 − 0.96)
logrank P = 0.021
Survival probability
1.0
0.8
0.6
0.4
0.2
0.0
Expression
low
high
0
50
100
150
200
Time (months)
Number at risk
FABP5low 935 406 88 10 2
FABP5high 401 197 44 5 1

Figure S5

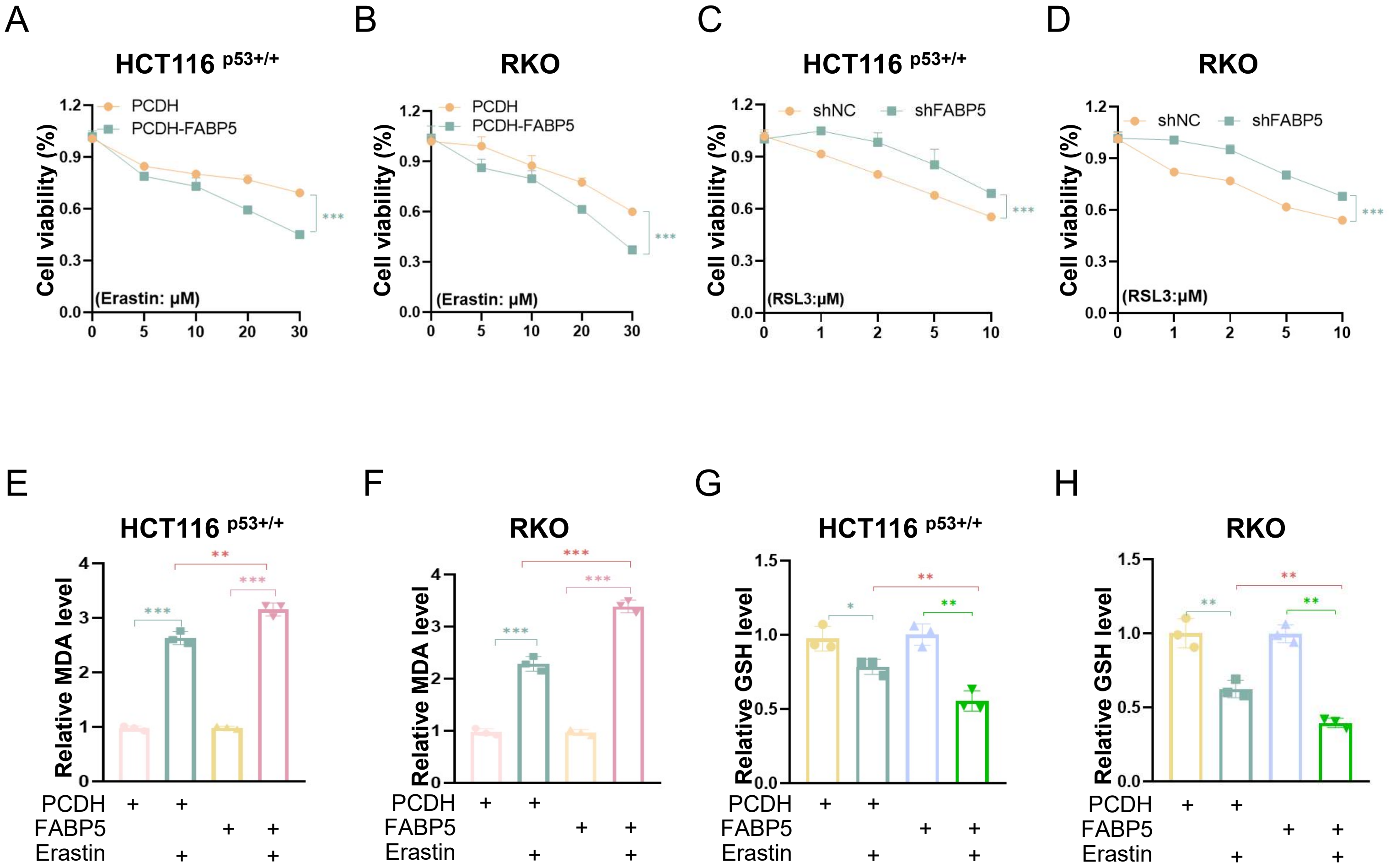
A
HCT116 p53+/+
PCDH
PCDH-FABP5
Cell viability (%)
(Erastin: μM)
B
RKO
PCDH
PCDH-FABP5
Cell viability (%)
(Erastin: μM)
C
HCT116 p53+/+
shNC
shFABP5
Cell viability (%)
(RSL3:μM)
D
RKO
shNC
shFABP5
Cell viability (%)
(RSL3:μM)
E
HCT116 p53+/+
Relative MDA level
F
RKO
Relative MDA level
G
HCT116 p53+/+
Relative GSH level
H
RKO
Relative GSH level
PCDH
FABP5
Erastin

Figure S6

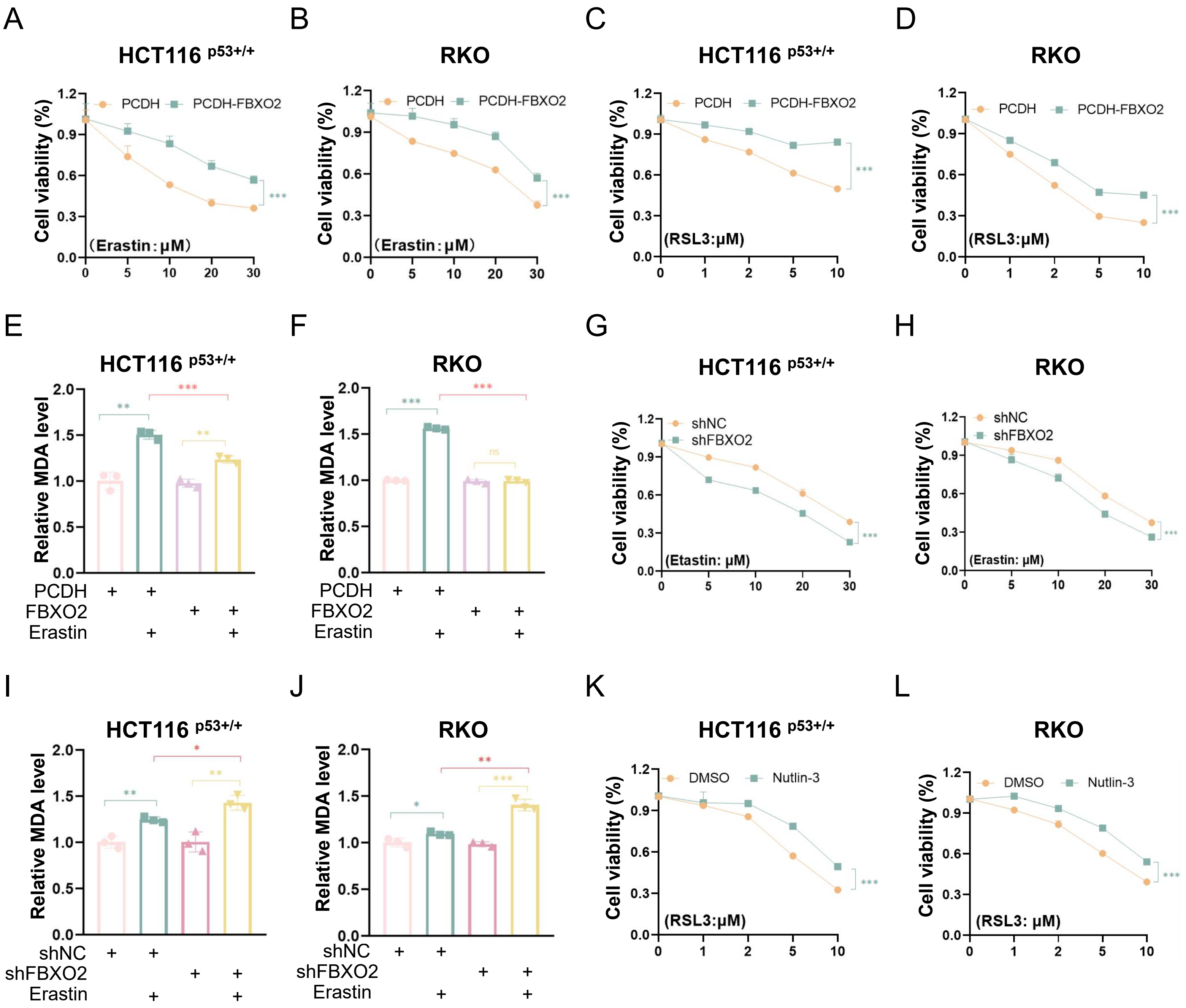
A
HCT116 p53+/+
PCDH
PCDH-FBXO2
Cell viability (%)
(Erastin : μM)
B
RKO
(Erastin : μM)
C
HCT116 p53+/+
(RSL3:μM)
D
RKO
(RSL3:μM)
E
HCT116 p53+/+
Relative MDA level
PCDH
FBXO2
Erastin
F
RKO
ns
G
HCT116 p53+/+
shNC
shFBXO2
(Etastin: μM)
H
RKO
(Erastin: μM)
I
HCT116 p53+/+
shNC
shFBXO2
Erastin
J
RKO
K
HCT116 p53+/+
DMSO
Nutlin-3
(RSL3:μM)
L
RKO
(RSL3: μM)

Figure S7

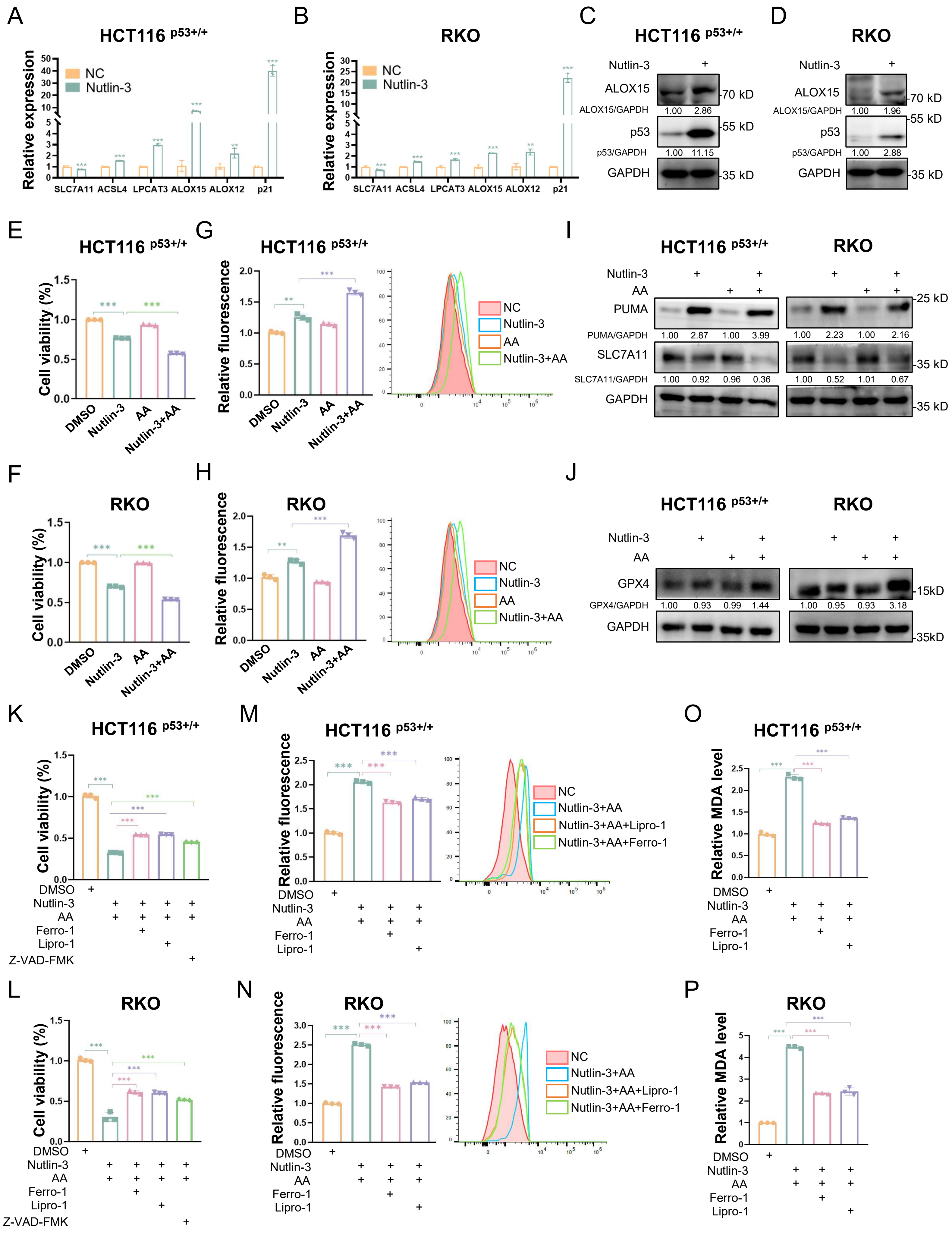

A
HCT116 p53+/+
Relative expression
NC
Nutlin-3
SLC7A11 ACSL4 LPCAT3 ALOX15 ALOX12 p21
B
RKO
C
HCT116 p53+/+
Nutlin-3
ALOX15
ALOX15/GAPDH 1.00 2.86
p53
p53/GAPDH 1.00 11.15
GAPDH
70 kD
55 kD
35 kD
D
RKO
ALOX15/GAPDH 1.00 1.96
p53/GAPDH 1.00 2.88
E
Cell viability (%)
DMSO
Nutlin-3
AA
Nutlin-3+AA
F
G
Relative fluorescence
H
I
PUMA
PUMA/GAPDH 1.00 2.87 1.00 3.99 1.00 2.23 1.00 2.16
SLC7A11
SLC7A11/GAPDH 1.00 0.92 0.96 0.36 1.00 0.52 1.01 0.67
25 kD
J
GPX4
GPX4/GAPDH 1.00 0.93 0.99 1.44 1.00 0.95 0.93 3.18
15kD
35kD
K
Ferro-1
Lipro-1
Z-VAD-FMK
L
M
Nutlin-3+AA
Nutlin-3+AA+Lipro-1
Nutlin-3+AA+Ferro-1
N
O
Relative MDA level
P

Figure S8

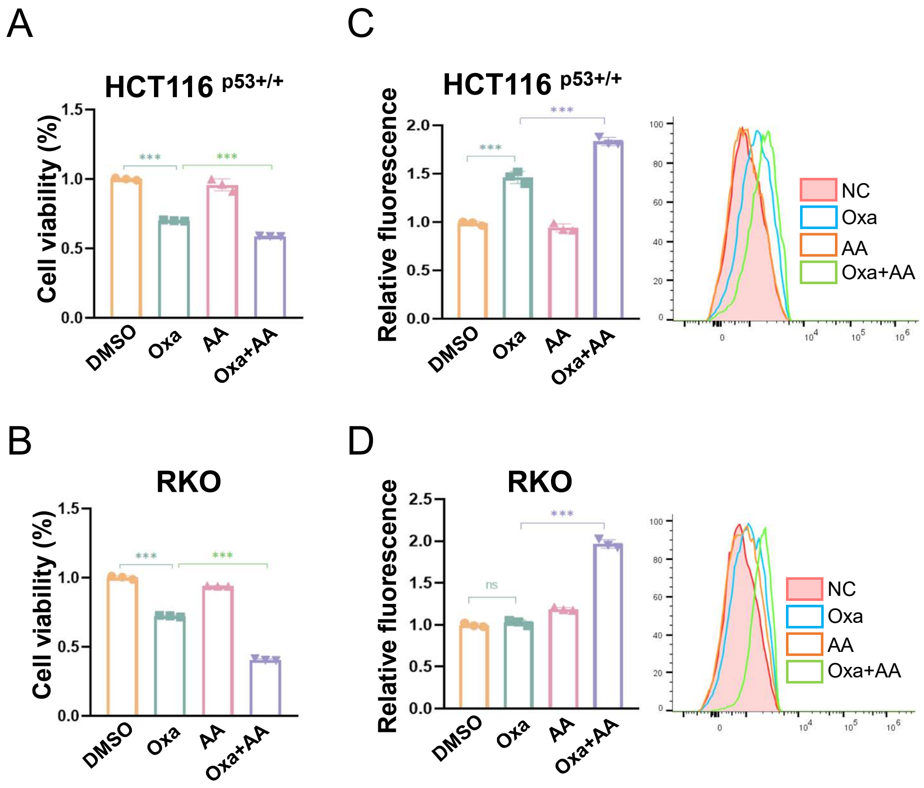
A
HCT116 p53+/+
Cell viability (%)
1.5
1.0
0.5
0.0
***
***
DMSO
Oxa
AA
Oxa+AA
B
RKO
Cell viability (%)
***
***
DMSO
Oxa
AA
Oxa+AA
C
HCT116 p53+/+
Relative fluorescence
2.0
1.5
1.0
0.5
0.0
***
***
DMSO
Oxa
AA
Oxa+AA
NC
Oxa
AA
Oxa+AA
D
RKO
Relative fluorescence
2.5
2.0
1.5
1.0
0.5
0.0
ns
***
DMSO
Oxa
AA
Oxa+AA
NC
Oxa
AA
Oxa+AA

Supplementary Tables

Supplementary Table S1. Univariate and multivariate Cox regression analysis of overall survival in patients with colorectal cancer

Characteristics	Total (N)	Univariate analysis (HR, 95% CI)	P value	Multivariate analysis (HR, 95% CI)	P value
**Gender**	90				
Male (Ref)	46	Reference			
Female	44	1.140 (0.607-2.142)	0.683 (PH=0.712)		
**Age**	90				
<60 (Ref)	26	Reference			
≥60	64	1.277 (0.649-2.513)	0.478 (PH=0.519)		
**T stage**	90				
T1-2 (Ref)	16	Reference			
T3-4	74	7.202 (1.723-30.095)	0.007 (PH=0.448)		
**N stage**	90				
N0 (Ref)	34	Reference			
N1-2	56	5.324 (2.331-12.160)	0 (PH=0.277)		
**TNM stage**	90				
I-II (Ref)	33	Reference			
III-IV	57	5.067 (2.218-11.578)	0 (PH=0.296)	3.684 (1.512-8.971)	0.004
**FBXO2**	90				
Low (Ref)	33	Reference			
High	57	2.934 (1.342-6.415)	0.007 (PH=0.349)	2.383 (1.063-5.342)	0.035
**FABP5**	90				
Low (Ref)	39	Reference			
High	51	0.406 (0.212-0.776)	0.006 (PH=0.705)	0.508 (0.260-0.992)	0.047
**p53**	90				
WT (Ref)	36	Reference			
MT	28	0.778 (1.285-0.706)	0.369 (PH=0.229)		
Unknown	26				

Supplementary Table S2. Primers for construction

Primer	Sequence (5'-3')
FBXO2-p53-RE-WT-F	GGGGTACCTTCTGGGACCATGTCAGTAGGCCCAA
FBXO2-p53-RE-WT-R	CCAAGCTTGCTGCGGAGGGCGGTCGCGAGAGGA
Flag-FBXO2-F	CGGAATTCAAATGGACGGAGACGGTGACCCA
Flag-FBXO2-R	GCTCTAGATCAGGGTTCTACCCACACGCTGCT
pCDNA-FBXO2-F	GCTCTAGAATGGACGGAGACGGTGA
pCDNA-FBXO2-R	CGGAATTCGGGTTCTACCCACACGCT
Flag-FBA-F	CGGAATTCTTTACTTCCTGAGCAAGCGGCGCCGCA
Flag-linker+FBA-F	CGGAATTCTTAAGTGCCAGCAGGAGGGGCTGGT
Flag-F-box+linker+FBA-F	CGGAATTCTTGCCGCGTACCTGGACGAGCT
PCDH-FABP5-F	GCTCTAGATGGCCACAGTTCAGCAGCT
PCDH-FABP5-R	CGGAATTCTTTATCGTCATCGTCTTTGTAGTCTT
pCDNA-FABP5-F	GCTCTAGAATGGCCACAGTTCAGCAGCTGGA
pCDNA -FABP5-R	CGGAATTCGTTCTACTTTTTCATAGATCCGAGT
Flag-FABP5-F	CGGAATTCATGGCCACAGTTCAGCAGCT
Flag-FABP5-R	GCTCTAGATTATTCTACTTTTTCATAGATCCG
PCMV-CUL1-F	TACGCTCTTATGGCCACCGGTA
	TGTCGTCAACCCGGAGCC
PCMV-CUL1-R	GATCCCCGCGGCCGCGGTACCAG
	CCAAGTAACTGTAGGTGTCCTTT

Supplementary Table S3. Primers for qPCR

Primer	Sequence (5'-3')
GAPDH-F	GGAGCGAGATCCCTCCAAAAT
GAPDH-R	GGCTGTTGTCATACTTCTCATGG
ACTB-F	CATGTACGTTGCTATCCAGGC
ACTB-R	CTCCTTAATGTCACGCACGAT
p53-F	CCCAAGCAATGGATGATTTGA
p53-R	GGCATTCTGGGAGCTTCATCT
p21-F	CTGGACTGTTTTCTCTCGGCTC
p21-R	TGTATATTCAGCATTGTGGGAGGA
FBXO2-F	GTGTCGCAAAGCACAGGTC
FBXO2-R	CGGACAGTAGCTTAACGGTGAG
CHIP-FBXO2-F	AGTAGCTACCTCTTTGGGTTACT
CHIP-FBXO2-R	GACCCGGCCTGTACCCCACCTTCA
CHIP-P21-F	GCTCCCTCATGGGCAAACTCACT
CHIP-P21-R	TGGCTGGTCTACCTGGCTCCTCT
LAMP2A-F	GAAAATGCCACTTGCCTTTATGC
LAMP2A-R	AGGAAAAGCCAGGTCCGAAC
SLC7A11-F	GCGTGGGCATGTCTCTGAC
SLC7A11-R	GCTGGTAATGGACCAAAGACTTC
ACSL4-F	ACTGGCCGACCTAAGGGAG
ACSL4-R	GCCAAAGGCAAGTAGCCAATA
LPCAT3-F	GGAGACCTACCTCATCCACCT
LPCAT3-R	CGGCCCATTAGTCGAAGGA
ALOX12-F	GCTCCTGGAACTGCCTAGAA
ALOX12-R	TCATCATCCTGCCAGCACT
ALOX15-F	AGCCTGATGGGAAACTCTTG
ALOX15-R	AGGTGGTGGGGATCCTGT

Supplementary Table S4. Sequences for siRNAs

siRNA	Sequence (5'-3')
siNC	UUCUCCGAACGUGUCACGUTT
sip53	GUAAUCUACUGGGACGGAATT
siFBXO2-1	GGGTAGATAGGCCTTAACTATT
siFBXO2-2	CTTTGAGTGGTGTCGCAAAATT
siLAMP2A-1	GCAGUGCAGAUGACGACAATT
siLAMP2A-2	GCCUUGGCAGGAGUACUUATT
siFABP5-1	GGAATAGCTTTGCGAAAAATT
siFABP5-2	CAATGAGCAAATCTCCATATT

Supplementary Table S5. Primers for shRNA

Primer	Sequence (5'-3')
shFBXO2-1-F	CCGGCACCGTTAAGCTACTGTCCGACTCGAG TCGGACAGTAGCTTAACGGTGTTTTTG
shFBXO2-1-R	AATTCAAAAACACCGTTAAGCTACTGTCCGACTCGAG TCGGACAGTAGCTTAACGGTG
shFBXO2-2-F	CCGGTCGTGGTGAAGGACTGGTACTCTCGAG AGTACCAGTCCTTCACCACGATTTTTG
shFBXO2-2-R	AATTCAAAAATCGTGGTGAAGGACTGGTACTCTCGAG AGTACCAGTCCTTCACCACGA
shFABP5-F	CCGGTGGGAAGGAAAGCACAATACTCG AGTATTGTGCTTTCCTTCCCATTTTTG
shFABP5-R	AATTCAAAAATGGGAAGGAAAGCACAATA CTCGAGTATTGTGCTTTCCTTCCCA
